# Advancing Biomaterials Evaluation: A Human Quadruple Bone Cell Culture Reveals Molybdenum-Driven Pro-Osteogenic and Anti-osteoclastogenic Responses

**DOI:** 10.1021/acsami.6c04674

**Published:** 2026-05-07

**Authors:** Katharina Wirsig, Anne Bernhardt

**Affiliations:** Centre for Translational Bone, Joint- and Soft Tissue Research, Faculty of Medicine and University Hospital Carl Gustav Carus, TUD University of Technology, Fetscherstraße 74, 01307 Dresden, Germany

**Keywords:** bone, molybdenum, coculture, osteoblast, osteocyte, osteoclast, biomaterial

## Abstract

The increasing prevalence of bone-related diseases and the desire to improve patient outcomes are driving research into bone replacement materials that overcome the limits of current bone substitutes. Molybdenum (Mo) is a promising candidate as an implant and degradable bone replacement material because it combines three key properties: mechanical strength, biocompatibility, and resorbability. However, little is known about the cellular mechanisms induced by Mo on bone regeneration. This study exposed a complex in vitro bone model as quadruple culture with primary human osteoblasts, osteocytes, osteoclasts, and endothelial cells, to Mo powder extracts to understand cell-material interactions in a multicellular system. Extracts with a final concentration of 1 mM Mo in quadruple cultures induced osteogenic differentiation by stimulation of *ALPL* gene expression and ALP activity, *BMP-2* and *BGLAP* gene expression, as well as enhanced calcium deposition of osteoblasts. Furthermore, *VEGFA* expression of osteoblasts increased significantly and network formation of HUVEC with stimulated *VWF* expression occurred. However, CD31 (*PECAM1*) expression and endothelial network density were reduced, indicating a complex, mixed angiogenic response. In contrast, Mo inhibited osteoclast formation and slowed down osteocyte differentiation, reducing *SOST*, *DMP1*, and *MEPE* gene expression. Additionally, the RANKL (*TNFSF11*)/OPG (*TNFRSF11B*) ratio of osteocytes was shifted toward OPG after Mo treatment. Cellular effects are most likely caused by the presence of molybdate anions. In summary, Mo extracts stimulated early bone healing factors involved in osteogenesis, vascularization, and mineralization, while osteoclastogenesis was inhibited. These dual effects in vitro provide mechanistic evidence supporting the potential of Mo as a growth factor-free bone replacement material and establish a cellular foundation for further preclinical development.

## Graphical abstract

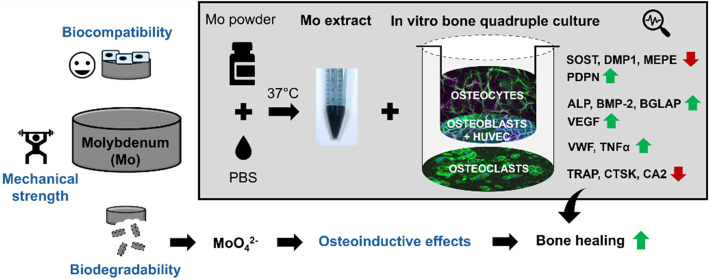

## 1 Introduction

The use of animal models is a common practice to investigate physiological and pathological cellular processes in bone, as well as to develop new therapeutic interventions. However, conventional animal models frequently fail to replicate human-specific bone physiology, leading to challenges in translating preclinical findings to clinical applications.^1^ As recently reported, only 5% of animal tested therapies obtain regulatory approval for human applications.^2^ Consequently, there is a growing emphasis on developing new approach methodologies (NAMs), including in silico, ex vivo, but also human-based in vitro models that accurately mimic the cellular complexity and functionality of native tissues, like bone, to advance regenerative medicine and reduce reliance on costly and ethically questionable animal testing.^3^

The architecture and function of bone tissue are dynamically regulated by complex interactions and signaling pathways between bone-forming osteoblasts (OB) and bone-resorbing osteoclasts (OC), orchestrated by signal-transducing osteocytes (OCY). OCY develop dendritic outgrowths that form a three-dimensional network deeply embedded in the bone matrix. This network enables OCY to sense biochemical or mechanical stimuli and subsequently convert them into signals that influence the activity of OB or OC located on the bone surface.^4^ Bone tissue is highly vascularized, ensuring the supply of oxygen and nutrients, allowing for the migration of cells, and facilitating the removal of metabolic waste products. In addition, osteogenesis and angiogenesis are closely coupled processes during bone remodeling.^5–7^ OB and OCY secrete vascular endothelial growth factor (VEGF), which is crucial for recruiting endothelial progenitor cells, their differentiation, and the formation of blood vessels.^8^ In turn, endothelial cell (EC)-derived factors can induce osteogenesis.^9^ The complex crosstalk in bone tissue includes numerous of such signaling cascades, one of which is the RANK-RANKL-OPG axis.^10^ The receptor activator of NF-κB (RANK) is located on the surface of OC progenitor cells. OB and OCY produce both receptor activator of NF-κB ligand (RANKL) and its counteracting protein, osteoprotegerin (OPG).^11^ Binding of RANKL to its receptor, RANK, induces the differentiation and resorptive activity of OC, while OPG blocks this binding, following inhibition of osteoclastogenesis. An in vitro bone model for studying the bone metabolism, including cellular crosstalk, should therefore involve both bone cells and EC. Recent advancements have led to the development of sophisticated three-dimensional (3D) coculture systems with multiple cell types. However, these systems hold some drawbacks. First, cell lines and animal-derived cells are often used instead of primary human cells.^12,13^ Second, triple or quadruple cultures include a mixture of different cell types excluding separate analysis of each cell type.^14,15^ To overcome these limitations, an in vitro bone model as quadruple culture with primary human OB, OCY, OC, and human umbilical vein endothelial cells (HUVEC) as transwell insert construct was recently established.^16^ This bone model enables simultaneous differentiation of OCY and OC by cellular crosstalk, without the need for external addition of RANKL and macrophage-colony stimulating factor (M-CSF). Additionally, separate analyses of OCY, OC, and OB + HUVEC at morphological, gene expression, protein secretion, and enzyme activity levels allow the investigation of osteogenesis, osteoclastogenesis, angiogenesis, and mineralization in parallel in a controlled environment. Therefore, this model is suitable to study cellular effects of drugs or biomaterial extracts on bone metabolism in vitro.

The need to develop new bone replacement materials results from the limited existing options. Autografts, while considered the gold standard, are limited by available bone volume and donor site morbidity, and allografts carry the risk of disease transmission.^17^ Synthetic materials developed through bone tissue engineering, including techniques like 3D printing, often do not perfectly mimic the native bone structure or promote optimal bone regeneration. Molybdenum (Mo) is an essential trace element which is part of cofactors required for several mammalian enzymes,^18^ and is also involved in sulfur and iron metabolism.^19^ The bioavailable and metabolically active form for organisms is the molybdate anion (MoO_4_^2–^).^20^ Mo combines three important properties: mechanical strength, biocompatibility,^21^ and biodegradability.^22,23^ These properties make the metal a promising and increasingly recognized candidate as novel biomaterial for bone regeneration. Mo could provide structural support during bone healing and gradually degrade as new bone tissue grows into and around the material. This resorbability is a major advantage of Mo compared to traditional nonresorbable materials, such as titanium, which require surgical removal. In initial trials, Schauer et al. observed degradation of Mo wires in vitro (33.6 μm/year) and in vivo after implantation into the abdominal aorta of Wistar rats (13.5 μm/year).^22^ Additionally, implantation of Mo into bone defects in the skull area of domestic pigs showed early signs of degradation after 54 days.^24^ However, longer in situ residence times, as well as the implant design, especially in regard to porosity, could increase in vivo degradation rates.

Furthermore, Mo could be applied as a pure metallic implant, alloyed with other materials,^25^ or incorporated into 3D-printed scaffolds. On the biological side, there is growing evidence for Mo’s ability to promote bone growth and regeneration. Tian et al. published findings on pro-osteogenic and anti-osteoclastogenic effects of a 3D-printed Mo-containing scaffold on bone marrow mesenchymal stem cells and osteoclast progenitors.^26^ Additionally, osteogenic differentiation and formation of extracellular matrix (ECM) were stimulated by Mo-containing mesoporous bioactive glass nanoparticles.^27^ However, especially cellular effects of Mo on human EC and OCY are not yet comprehensively studied. To further characterize the biological effects of Mo on bone remodeling, the present study exposed the aforementioned advanced in vitro quadruple culture with primary human OB, OCY, OC, and HUVEC, to Mo extracts to evaluate the potential use of Mo in bone replacement materials.

## 2 Experimental Section

### 2.1 Cell Culture

Primary human pre-OB were obtained from femoral heads of osteoarthritic patients undergoing total hip replacement, as well as from knee femoral condyles during total knee joint replacement (arthroplasty) at the University Hospital *Carl Gustav Carus* Dresden (Germany) as previously described.^28^ Tissue collection was performed after informed consent and with approval by the ethics commission of TU Dresden (EK 303082014 and BO-EK-235052022). Briefly, bone tissue was minced and digested in a two-step process using a collagenase II solution. The resulting pre-OB fraction corresponds to the previously termed “control cells” by Bernhardt et al. Pre-OB were expanded in α-MEM with GlutaMAX (Gibco) containing 10% FCS, 100 U/mL penicillin and 100 μg/mL streptomycin (PS) (Gibco) up to passage 4 (Table 1).

**Table 1:** Media Formulations for Quadruple Culture Experiments^a^

Purpose	Media composition
**Expansion OB**	10% FCS, 1% PS in α-MEM
**Expansion HUVEC**	EGM (Promocell)
**Osteogenic differentiation OB**	**Expansion OB** + 10 mM β-GP, 12.5 μg/mL AAP, 10^–7^ M Dex
**Expansion PBMC**	10% hi FCS, 25 ng/mL MCSF, 1% PS in α-MEM
**Differentiation OC**	5% hi FCS, 5% HS, 25 ng/mL MCSF, 50 ng/mL RANKL, 1% PS in α-MEM
Basal medium quadruple culture **(BM quadruple)**	2% hi FCS, 5 mM β-GP, 12.5 μg/mL AAP, 20 ng/mL VEGF, 100 ng/mL BMP-2, 1% PS in 50% α-MEM/50% ECBM
**Quadruple culture**	BM quadruple + 1 mM Mo

a Fetal calf serum (FCS), penicillin/streptomycin (PS), endothelial cell growth medium (EGM), β-glycerophosphate (β-GP), ascorbic acid-2-phosphate (AAP), dexamethasone (Dex), heat inactivated FCS (hi FCS), human serum (HS), macrophage colony stimulating factor (MCSF), receptor activator of NF-κB ligand (RANKL), endothelial cell basal medium (ECBM), vascular endothelial growth factor (VEGF), bone morphogenetic protein 2 (BMP-2).

To induce osteogenic differentiation, pre-OB in passage 4 were cultivated for 7 days in osteogenic medium consisting of expansion medium further supplemented with 10^–7^ M dexamethasone (Dex), 10 mM β-glycerophosphate (β-GP) and 12.5 μg/mL ascorbic acid-2-phosphate (AAP) (all osteogenic supplements from Sigma-Aldrich). For OB monoculture, 1 × 10^4^ OB per well were seeded into 48-well plates in expansion medium. After three days, medium was replaced either with control medium (OB expansion medium) or with OB expansion medium containing varying concentrations of a Mo extract. OB were incubated for 14 days, with a medium change after seven days.

For OCY differentiation, predifferentiated OB were embedded into a collagen gel as described before.^29^ Briefly, a mixture consisting of eight parts collagen solution (4 mg/mL rat tail collagen, Meidrix Biomedicals GmbH, dissolved in 0.1 M acetic acid) and one part of 10× HBSS was neutralized with 1 N NaOH and supplemented with 10 mM β-GP and 12.5 μg/mL AAP. OB were suspended in the collagen gel at a density of 1 × 10^5^ cells/mL and filled into transwell inserts. Fifferentiation into OCY was achieved over a 14-day culture period, with the addition of 100 ng/mL BMP-2 to promote expression of late OCY markers.^16,30^

HUVEC (Promocell, Cat. number: C-12205) were expanded in endothelial cell growth medium (EGM, Promocell) with medium renewal every three to four days until passage 3.

OC were differentiated from peripheral blood mononuclear cells (PBMC), which were isolated from human buffy coat concentrates (German Red Cross Dresden) by density gradient centrifugation, as previously descrobed.^31^ For subsequent quadruple culture experiments, 1.5 × 10^6^ PBMC per 24-well were initially incubated in PBMC expansion medium (Table 1) until assembly of quadruple cultures. For OC monoculture conditions, 5 × 10^5^ PBMC were seeded in 48 well plates in PBMC expansion medium. After three days, medium was replaced to OC differentiation medium supplemented with MCSF and RANKL (Table 1). Medium was changed after 3 days, and OC differentiation was completed after 7 days. For predifferentiation of OC, 1 × 10^7^ PBMC were seeded into 25 cm^2^ culture flasks pretreated with antiadhesion solution (Stem Cell Technologies), and maintained in OC differentiation medium for 7 days. Subsequently, multinucleated OC were detached by incubation with 2.5 mM EDTA in PBS for 20 min and used for cytotoxicity tests.

### 2.2 Quadruple Cultures

Bone quadruple cultures incorporating primary human OB, OCY, OC and HUVEC were established utilizing transwell inserts (0.4 μm pore size, cellQART) in 24-well plates, following a protocol described in our prior work^16^ (Figure 1). OCY differentiation from collagen gel embedded OB^29^ occurred concurrently with OC formation from PBMC.^16,31^ Briefly, OB in passage 4, following osteogenic differentiation, were detached with Trypsin/EDTA (Gibco) and seeded onto the basal side of transwell insert membranes at a density of 1 × 10^4^ cells per membrane by inverting the inserts. Cell adhesion was facilitated by a 1 h incubation at 37 °C without further medium supply. Subsequently, transwell inserts were placed in 24-well plates containing OB expansion medium. Following a three-day incubation period at 37 °C, HUVEC in passage 4 were seeded on top of the OB layer on the basal side of the insert membrane at a density of 1 × 10^4^ cells per membrane. They were allowed to adhere for 30 min. Afterward, predifferentiated OB were embedded in a collagen gel matrix to facilitate OCY differentiation as outlined above. Per sample, 300 μL of the collagen-cell suspension were transferred to the apical side of the transwell inserts (upper part). Collagen gelation was achieved through a 30 min incubation at 37 °C. Constructs were then placed in 24-well plates, preseeded with PBMC three days prior. Upon completion of quadruple culture assembly, medium was added according to the formulation detailed in Table 1. Quadruple culture medium consisted of a 1:1 mixture of α-MEM (Gibco) and endothelial cell basal medium (ECBM; Promocell), supplemented with 2% heat inactivated (hi) FCS, 1% PS, osteogenic supplements β-GP and AAP, BMP-2 (recombinant human BMP-2/InductOs; Medtronic) to support stable OCY differentiation, and VEGF (recombinant human VEGF_165_; PeproTech) to promote endothelial cell tube formation.^16^ A Mo-free control group cultured in basal quadruple culture medium (BM quadruple; Table 1) was compared to a 1 mM Mo-treated group. Quadruple cultures were maintained at 37 °C for 14 days, with a medium change after 7 days. Control conditions were designed to ensure the concurrent differentiation of human OCY and OC alongside HUVEC network formation in the quadruple culture system. The behavior of all involved cell types under these control conditions has been comprehensively characterized with respect to morphology, gene expression, enzymatic activity, and protein secretion, as reported in our previous work.^16^

**Figure 1:**
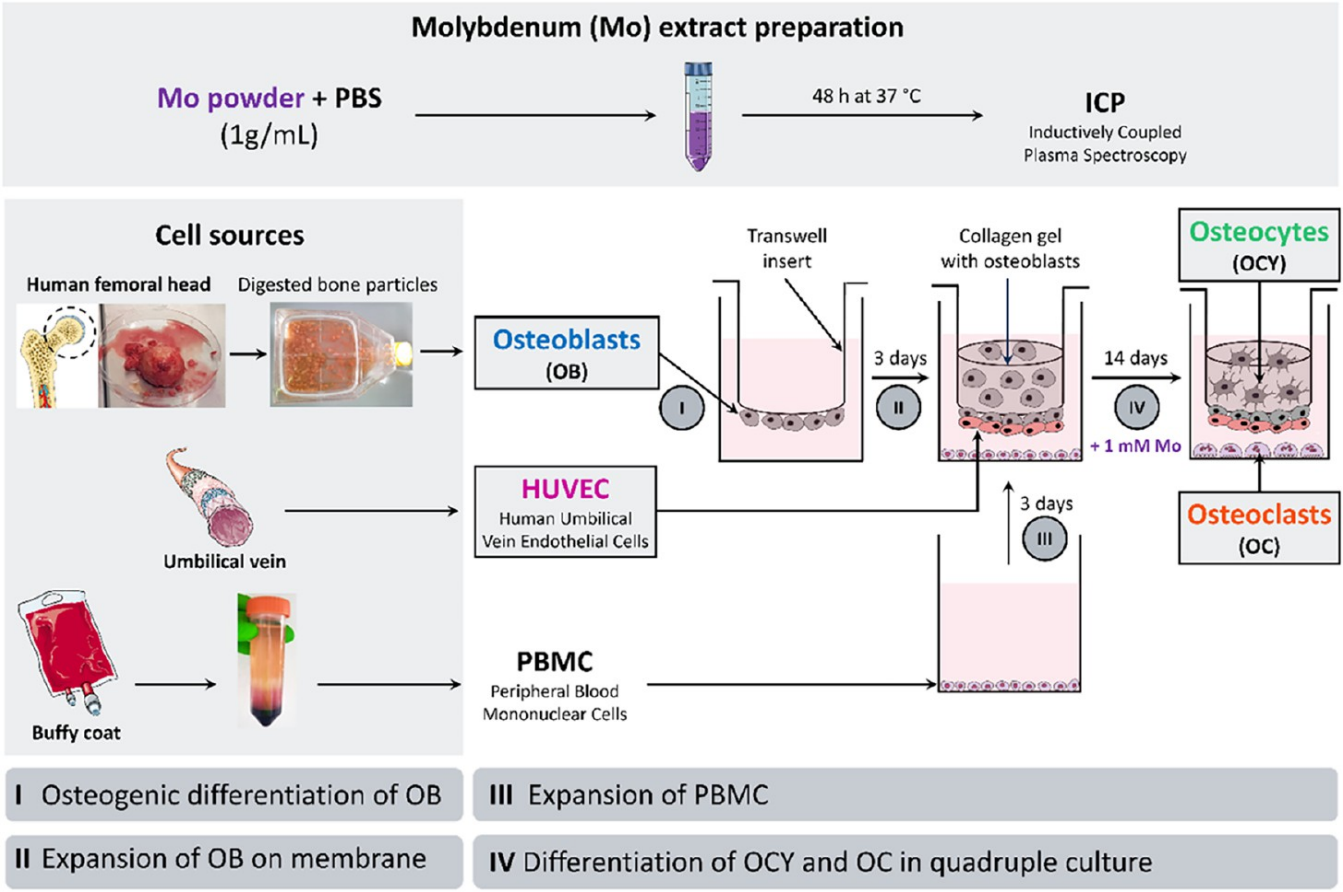
Schematic representation of the experimental setup for bone quadruple cultures comprising primary human OB, OCY, OC, and EC, assembled as transwell insert constructs. A Mo extract was obtained after incubating a mixture of Mo powder and PBS for 48 h at 37 °C and subsequent centrifugation. OB were obtained from human femoral heads or femoral condyles. PBMC were isolated from human buffy coat by density gradient centrifugation. HUVEC were co-seeded with OB onto the basal side of transwell insert membranes. OB embedded in collagen gel were subsequently deposited onto the apical side of the transwell inserts. Constructs were placed in well plates preseeded with PBMC. Over 14 days, collagen gel-embedded OB differentiate into OCY, and OC differentiate simultaneously from PBMC. This figure was adapted from Wirsig et al.^16^ Available under a CC BY 4.0 license. Copyright Wirsig et al. It includes modified illustrations sourced from Servier Medical Art (Servier; https://smart.servier.com/), licensed under a Creative Commons Attribution 4.0 unported license.

Four independent quadruple culture experiments were conducted, each with distinct donor combinations comprising OB/OCY derived from three different donors, HUVEC from two donors, and PBMC from three donors (Table 2). Information on age and gender of HUVEC and PBMC donors was not provided. Quadruple cultures were not personalized; rather, they were derived from cell types isolated from distinct, unrelated donors. OB and OCY always originate from the same donor, while PBMC and HUVEC originate from different donors.

**Table 2:** Experimental Groups and Donor Information of Four Individual Quadruple Culture Experiments with Different Donor Combinations

Quadruple culture Exp. Nr.	Donor combination	Donor information OB/OCY	Tissue
Exp. 1	hOB/hOCY 1	Female	Femoral head
	HUVEC 1	58 years	
	PBMC 1		
Exp. 2	hOB/hOCY 2	Female	Knee femur condyle and tibia plate
	HUVEC 1	62 years	
	PBMC 1		
Exp. 3	hOB/hOCY 2	Female	Knee femur condyle and tibia plate
	HUVEC 1	62 years	
	PBMC 2		
Exp. 4	hOB/hOCY 3	Male	Knee femur condyle and tibia plate
	HUVEC 2	65 years	
	PBMC 3		

### 2.3 Molybdenum Extract Preparation

For technical reasons of applicability, a Mo extract was tested in bone quadruple cultures instead of solid metallic Mo samples, allowing cell-material (extract) interactions with all cell types involved in the in vitro model. Therefore, Mo powder (spherical, −170 mesh; Thermo scientific) was incubated in PBS with a powder to liquid ratio of 1 g/mL for 48 h at 37 °C on a shaker. Then, the extract was centrifuged for 5 min at 1500 rpm, and the supernatant was collected. Mo concentration in the extract was quantified using inductively coupled plasma optical emission spectroscopy (ICP-OES) (Plasma Quant Elite, Analytik Jena) with respect to a calibration line composed from a commercially available Mo standard solution (Merck, TraceCERT), revealing a Mo concentration of ∼50 mM. For cell culture experiments, Mo extracts were diluted to concentrations ranging from 0.05 to 5.0 mM Mo in the respective culture medium.

### 2.4 Cytotoxicity Assay

Primary human OB or HUVEC were seeded in their respective expansion medium (Table 1) in white 96-well plates at a density of 1 × 10^3^ cells per well. Similarly, 1 × 10^4^ PBMC or predifferentiated OC, corresponding to an initial PBMC number of 5 × 10^5^ per well, were seeded in PBMC expansion medium or OC differentiation medium in white 96-well plates. For each cell type and donor, triplicates were seeded. After 24 h, medium was changed to the respective culture medium supplemented with Mo extracts at concentrations ranging from 0 to 5.0 mM. After another 24 h, cell viability was assessed using the commercially available CellTiterGlo luminescent assay (Promega) according to manufacturer’s instructions.

### 2.5 Fluorescence Microscopy

After cultivation, transwell insert membranes with OB and HUVEC were carefully detached from transwell inserts using surgical knife and tweezers. OCY-containing collagen gels were likewise removed from the inserts. All compartments, membranes with OB + HUVEC, OCY in collagen gels, and OC in well plates, were subjected to sequential washing (PBS), fixation (4% formaldehyde in PBS), permeabilization (0.1% Triton X-100 in PBS for 5 min, followed by PBS washing), and blocking (1% BSA in PBS for 30 min).

For immunofluorescence staining, OB + HUVEC membranes were incubated with mouse antihuman CD31 (1 μg/mL; Dako; Clone JC70A), while OCY were incubated with either rabbit antihuman DMP1 (3.3 μg/mL; TaKaRa; M176), rabbit antihuman SOST (100 μg/mL; antibodies-online GmbH; ABIN1714867) or mouse antihuman PDPN (1 μg/mL; antibodies-online GmbH; ABIN6939085), each applied overnight. After washing, samples were incubated with 1 μg/mL DAPI, Phalloidin-iFluor 488 (Abcam) as well as 4 μg/mL AlexaFluor 546 goat antimouse IgG (Invitrogen; Lot: 1904466) for CD31 and PDPN detection, or 8 μg/mL AlexaFluor 546 goat antirabbit IgG (Invitrogen; Lot: 2423676) for DMP1 and SOST detection. All antibodies and fluorescent dyes were prepared in 1% BSA in PBS. OB cultures were stained with DAPI/Phalliodin-iFluor488 accordingly. Z-stack images were captured with a Keyence BZ-X810 fluorescence microscope, and image processing was conducted using ImageJ (Fiji).

### 2.6 Alizarin Red Staining

Calcium deposition as a measure of mineralization was evaluated by alizarin red staining. After 14 days in quadruple culture, transwell insert membranes bearing OB and HUVEC were separated from inserts using surgical knife and tweezers and fixed in 4% formaldehyde in PBS for 1 h. Samples were rinsed with distilled water and subsequently stained with 2% alizarin red dissolved in distilled water (pH 4.1–4.3; Sigma Aldrich) for 3 min. Following a final washing step, brightfield images were obtained using a Keyence BZ-X810 microscope and processed with ImageJ (Fiji).

### 2.7 TRAP Staining

OC were washed with PBS and fixed with 4% formaldehyde in PBS for 15 min at room temperature. Tartrate-resistant acid phosphatase (TRAP) activity was visualized by incubating samples for 30 min at 37 °C in a staining solution composed of 0.3 mg/mL Fast Red Violet LB (Sigma-Aldrich) dissolved in an aqueous buffer containing 0.05 M sodium acetate (Sigma-Aldrich), 0.05 M acetic acid (Sigma-Aldrich), 0.03 M sodium tartrate (Roth), 0.1 mg/mL naphthol AS-MX phosphate disodium salt (Sigma-Aldrich), and 0.1% Triton X-100 (Sigma-Aldrich). Samples were subsequently washed with PBS, and cell nuclei were counterstained for 10 min using Mayers Haemalaun solution (AppliChem), followed by rinsing in tap water. Brightfield images were captured using a Keyence BZ-X810 microscope.

### 2.8 RNA Isolation, cDNA Synthesis, and PCR

PET membranes with OB and HUVEC were cut of the transwell inserts using surgical knife and tweezers. Collagen gels containing OCY were removed from inserts and subjected to enzymatic digestion in collagenase II solution (3 mg/mL collagenase II in α-MEM, 10% FCS, 3 mM CaCl_2_, and 1% PS) for 1 h at 37 °C. Digests were washed with PBS and centrifuged to obtain OCY pellets. To obtain sufficient RNA quantities for reliable downstream analysis, lysates from four quadruple culture samples per experiment and treatment condition were used to isolate RNA of membranes with OB + HUVEC, OCY pellets, and OC in well plates using the commercially available E.Z.N.A. MicroElute Total RNA Kit (Omega Biotek). Lysates from two samples were pooled prior RNA extraction to finally obtain two RNA samples per group. While this step reduces biological resolution, it is necessary to obtain a sufficient amount of RNA for reliable PCR analysis.

Complementary DNA synthesis (cDNA synthesis) was carried out using the High-Capacity cDNA Reverse Transcription Kit (Applied Biosystems) in accordance with the manufacturer’s instructions.

Quantitative gene expression analysis was performed via qPCR using the TaqMan Fast Advanced Master Mix (Applied Biosystems) in combination with TaqMan Gene Expression Assays (all Applied Biosystems) according to manufacturer’s instructions. Table 3 provides a list of analyzed genes and the product specifications for the corresponding TaqMan Gene Expression Assays. PCR was run with an Applied Biosystems 7500 fast Real-Time PCR system. Expression levels were normalized to the stable expression of ACTB (actin β), and relative gene expression (fold change) was calculated using the 2^–ΔΔCt^ method.

**Table 3:** Genes, Their Abbreviation and Product Identification for the Respective TaqMan Gene Expression Assays Analyzed with RT-qPCR

Gene	Abbreviation	Product specification
Actin-β	*ACTB*	Hs01060665_g1
Alkaline phosphatase	*ALPL*	Hs01029144_m1
Bone γ-carboxyglutamate protein/Osteocalcin	*BGLAP*	Hs01587814_g1
Bone sialoprotein II	*IBSP*	Hs00173720_m1
Receptor activator of NF-κB ligand	*TNFSF11*	Hs00243522_m1
Osteoprotegerin	*TNFRSF11B*	Hs00900358_m1
Podoplanin	*PDPN*	Hs00366766_m1
Matrix extracellular phosphoglycoprotein	*MEPE*	Hs00220237_m1
Dentin matrix protein 1	*DMP1*	Hs01009391_g1
Sclerostin	*SOST*	Hs00228830_m1
Collagen type I α 1 chain	*COL1A1*	Hs00164004_m1
Bone morphogenetic protein 2	*BMP2*	Hs00154192_m1
Platelet endothelial cell adhesion molecule/CD31	*PECAM1*	Hs01065279_m1
Vascular endothelial growth factor	*VEGFA*	Hs00900055_m1
Vascular endothelial growth factor receptor 2/Kinase insert domain receptor	*KDR*	Hs00911700_m1
Von Willebrand factor	*VWF*	Hs01109446_m1
VE-cadherin	*CDH5*	Hs00174344_m1
Tartrate-resistant acid phosphatase	*ACP5*	Hs00356261_m1
Cathepsin K	*CTSK*	Hs00166156_m1
Carbonic anhydrase II	*CA2*	Hs01070108_m1

### 2.9 Quantification of Specific Enzyme Activities and DNA

ALP activity of OB in monoculture and quadruple cultures, as well as TRAP and CTSK activities of OC in quadruple cultures, were measured and normalized to the respective sample DNA content. For this purpose, OB monocultures in well plates, separated insert membranes with OB and HUVEC from quadruple cultures, or OC in well plates from quadruple cultures, were rinsed once with PBS and stored at −20 °C until further processing. Upon thawing, cell lysis was achieved by incubation in 1% Triton X-100 in PBS for 50 min, with an ultrasonication step for 10 min in between. ALP activity was assessed colorimetrically by the enzymatic conversion of the colorless substrate p-nitrophenyl phosphate to the yellowish product p-nitrophenol, and quantified by absorbance measurements at 405 nm.^32^

OC-specific enzyme activities were quantified as previously published.^33^ In brief, TRAP activity was determined via the cleavage of naphthol ASBI phosphate under acidic conditions in the presence of tartrate, with fluorescence detection at excitation and emission wavelengths of 405 and 520 nm, respectively. CTSK activity was quantified through the proteolytic cleavage of fluorogenic substrate Z-LR-AMC (Enzo Life sciences), monitored at excitation and emission wavelengths of 365 and 440 nm, respectively. All absorbance and fluorescence measurements were performed using an Infinite M200pro spectrofluorometer (Tecan Trading AG).

DNA concentration in cell lysates was measured using the Quantifluor One dsDNA kit (Promega), carried out in accordance with manufacturer’s instructions.

### 2.10 BGLAP, SOST, TNFα, and IL-1β ELISA

Cell culture supernatants were analyzed with the following ELISA kits according to the manufacturer’s protocol: Human Osteocalcin DuoSet ELISA #DY1419–05 and Human SOST/Sclerostin DuoSet ELISA #DY1406, both R&D Systems, using recombinant human BGLAP (312–10,000 pg/mL) or SOST (62.5–4000 pg/mL) calibration curves, as well as Human TNFα Standard TMB ELISA Development Kit (Catalog #900-T25) and Human IL-1β Standard TMB ELISA Development Kit (Catalog #900-T95), both PeproTech, using recombinant human TNFα (31.3–2000 pg/mL) or IL-1β (11.7–750 pg/mL) calibration curves.

### 2.11 Statistics

Quadruple cultures incorporating OB, OCY, OC, and HUVEC were conducted across four independent experiments, each utilizing different donor combinations to constitute biological replicates (Table 2). For each quadruple or monoculture condition, samples from individual experiments were seeded in triplicates for the measurement of cell type-specific enzyme activities, ELISA-based quantification of secreted proteins in cell culture supernatants, and CellTiterGlo luminescent assays. Gene expression analysis was performed in two RNA samples generated from four replicates for each experimental group. Morphological assessment was carried out in two replicates per condition and experiment. Therefore, a total of nine quadruple culture replicates were generated per condition and experiment, representing individual intradonor replicates (*n*), rather than technical replicates. Enzyme activity measurements, DNA quantification, ELISA, and PCR analyses were pipetted in technical duplicates per intradonor replicate or RNA sample. Statistical differences in gene expression were calculated at the level of ΔCt values. Group comparisons accounting for interexperiment variability arising from different donor combinations were conducted using two-way ANOVA followed by Šídák’s multiple comparisons test. GraphPad Prism 10.0 was used for all statistical analyses.

## 3 Results

### 3.1 Mo Is Cytocompatible for OB, HUVEC, OC, and PBMC up to a Concentration of 1 mM and Induced VEGF in OB Monocultures

To identify a cytocompatible Mo concentration for testing in bone quadruple cultures, first, OB monocultures were treated with diluted Mo extracts containing 0 to 5 mM Mo. After 24 h, OB viability was not reduced at concentrations up to 0.1 mM Mo (Figure 2A). In contrast, the highest concentration tested, 5 mM Mo, decreased OB viability to below 75% for both donors. Furthermore, after 14 days in monoculture, OB DNA content increased in a concentration-dependent manner up to 1 mM Mo, compared to the Mo-free control (Figure 2B). However, treatment with 2 mM Mo significantly decreased OB DNA content (*p* < 0.0001). Viability of HUVEC and PBMC was unaffected at concentrations up to 1 mM Mo (Figure 2C,D). A higher concentration of 2 mM Mo reduced viability of both cell types by 25% compared to the Mo-free control. Viability of mature OC remained stable up to 0.1 mM Mo (Figure 2E). However, an even higher Mo concentration of up to 2 mM allowed for OC viability of around or above 75% compared to the Mo-free control.

**Figure 2:**
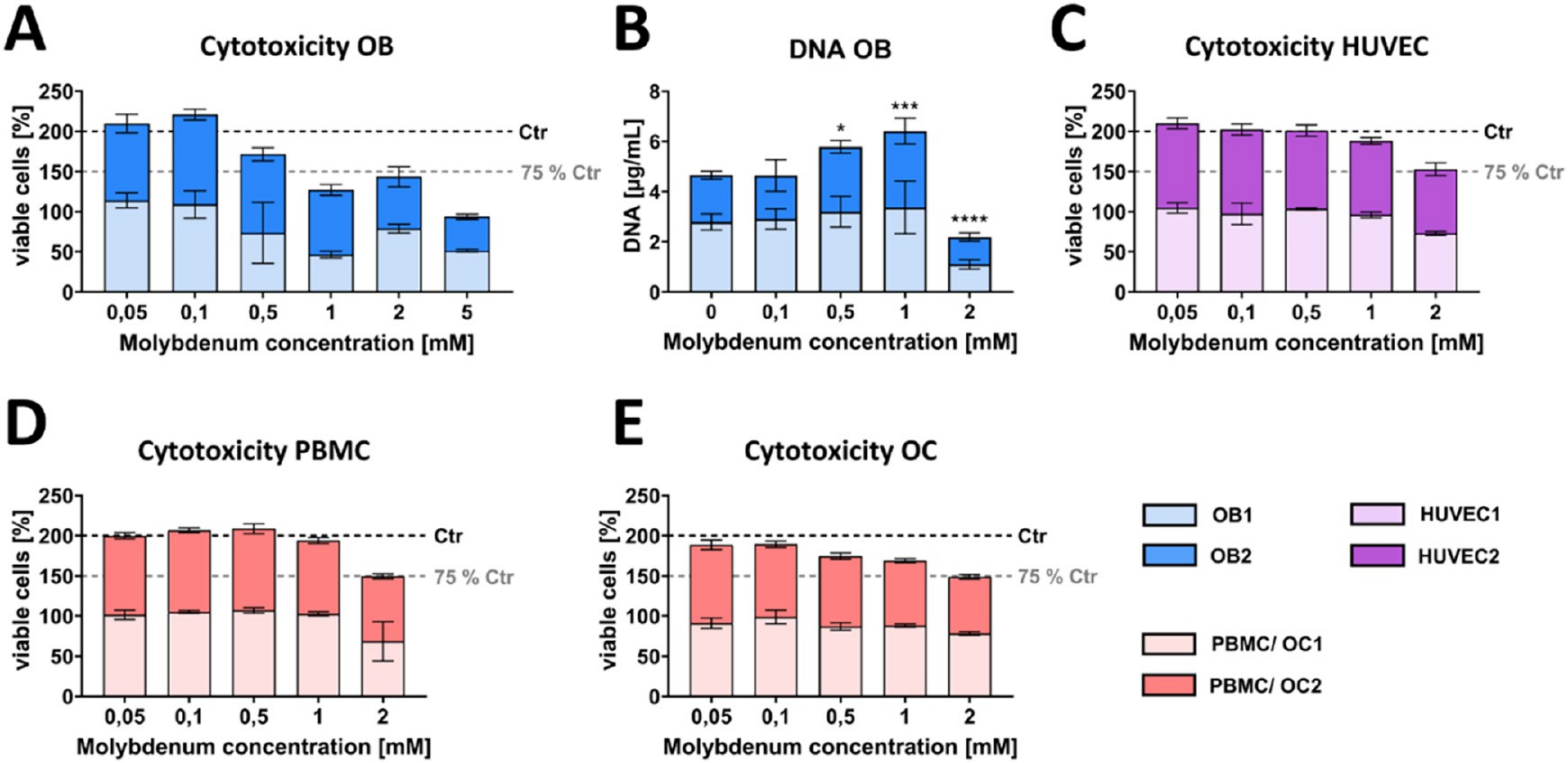
Cytotoxicity of Mo-extracts on OB, HUVEC, PBMC, and OC assessed by CellTiterGlo cell viability assay and DNA quantification. (A) Viability of OB derived from two donors in the presence of Mo ranging from 0.05 to 5 mM Mo compared to a Mo-free control after 24 h. (B) DNA amount of OB lysates after 14 days of Mo treatment with concentrations ranging from 0 to 2 mM. (C) Viability of HUVEC derived from two donors in the presence of Mo extract ranging from 0.05 to 2 mM Mo compared to a Mo-free control after 24 h. (D) Viability of PBMC derived from two donors in the presence of Mo extract ranging from 0.05 to 2 mM Mo compared to a Mo-free control after 24 h. (E) Viability of OC differentiated from PBMC derived from two donors in the presence of Mo extract ranging from 0.05 to 2 mM Mo compared to a Mo-free control after 24 h. Data are presented as mean ± standard deviation. Values exceeding 100% relative to the untreated control (0 mM Mo) are common in CellTiterGlo luminescent assays when treatment stimulate metabolic activity or proliferation. Each donor and condition *n* = 3 intradonor replicates. *p < 0.05: ***p < 0.001; ****p < 0.0001.

After 14 days treatment, morphology of OB in monocultures was not impaired at concentrations of up to 1 mM Mo for both donors (Figure 3A). OB showed an elongated, fibroblast-like morphology in all tested groups. However, cell density decreased visibly after 2 mM Mo treatment. In addition, gene expression of *COL1A1*, RANKL (*TNFSF11*), and *ALPL* strongly decreased in the presence of Mo in a concentration-dependent manner (Figure 3B). Accordingly, ALP activity of OB declined in the 1 mM and 2 mM Mo groups compared to the Ctr (Figure 3C). In contrast, *VEGFA* and OPG (*TNFRSF11B*) gene expression significantly increased after 1 mM Mo treatment (*p* < 0.0001). While strongest *VEGF* gene expression was observed in the 2 mM Mo group, OPG (*TNFRSF11B*) expression dropped with the highest Mo concentration tested. RANKL/OPG ratio was reduced in the 0.5 mM and 1 mM Mo groups, but increased after 2 mM Mo treatment (Figure 3D). *IBSP* gene expression was not detectable after 2 mM Mo treatment, and *BGLAP* expression showed no clear tendency for both OB donors.

**Figure 3:**
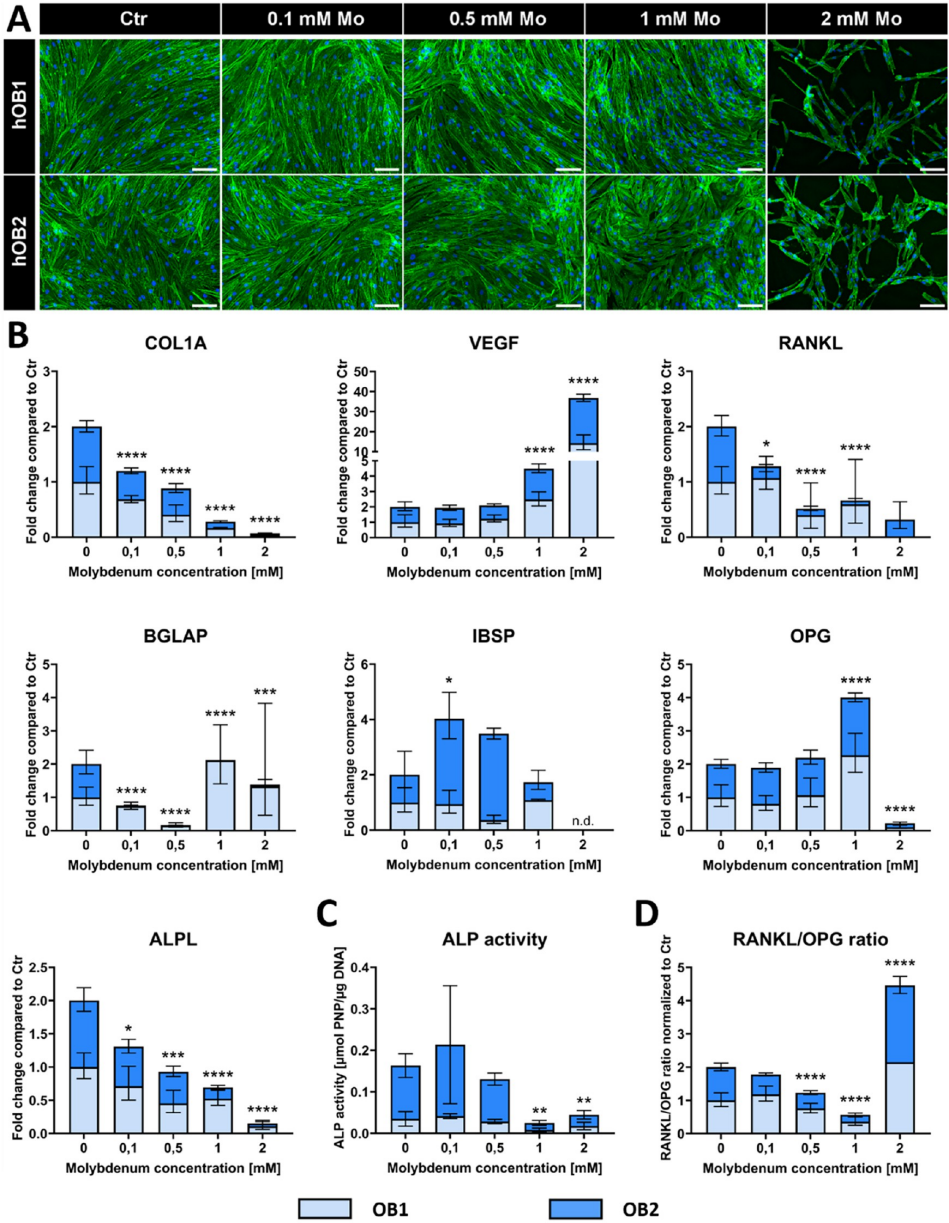
Impact of Mo extracts on OB in monoculture after 14 days treatment with different concentrations. (A) Fluorescence microscopic images. Cytoskeleton appears green (iFluor488 phalloidin) and cell nuclei appear blue (DAPI). Scale bars represent 100 μm. (B) Gene expression of OB markers presented as fold change normalized to Ctr (summary data of two experiments, each *n* = 4 intradonor replicates). (C) ALP activity of OB (summary data of two experiments, each *n* = 3 intradonor replicates). (D) RANKL/OPG ratio on gene expression level (summary data of two experiments, each *n* = 4 intradonor replicates). n.d. (not detectable). *p < 0.05; **p < 0.01; ***p < 0.001; ****p < 0.0001.

### 3.2 Mo Directly Inhibited OC Differentiation from PBMC in Monoculture

To analyze the direct effect of Mo on OC differentiation, PBMC were treated with different concentrations of a Mo extract in the presence of RANKL and MCSF. Without Mo (Ctr), rounded, multinucleated OC with intense TRAP staining differentiated from PBMC (Figure 4A,B). The presence of Mo extracts impaired OC differentiation in a concentration-dependent manner. Treatment with 0.5 mM Mo extract visibly decreased the size and number of multinucleated OC. In the 1 mM Mo group, OC formation was inhibited, resulting in the presence of mononuclear, elongated progenitors.

**Figure 4:**
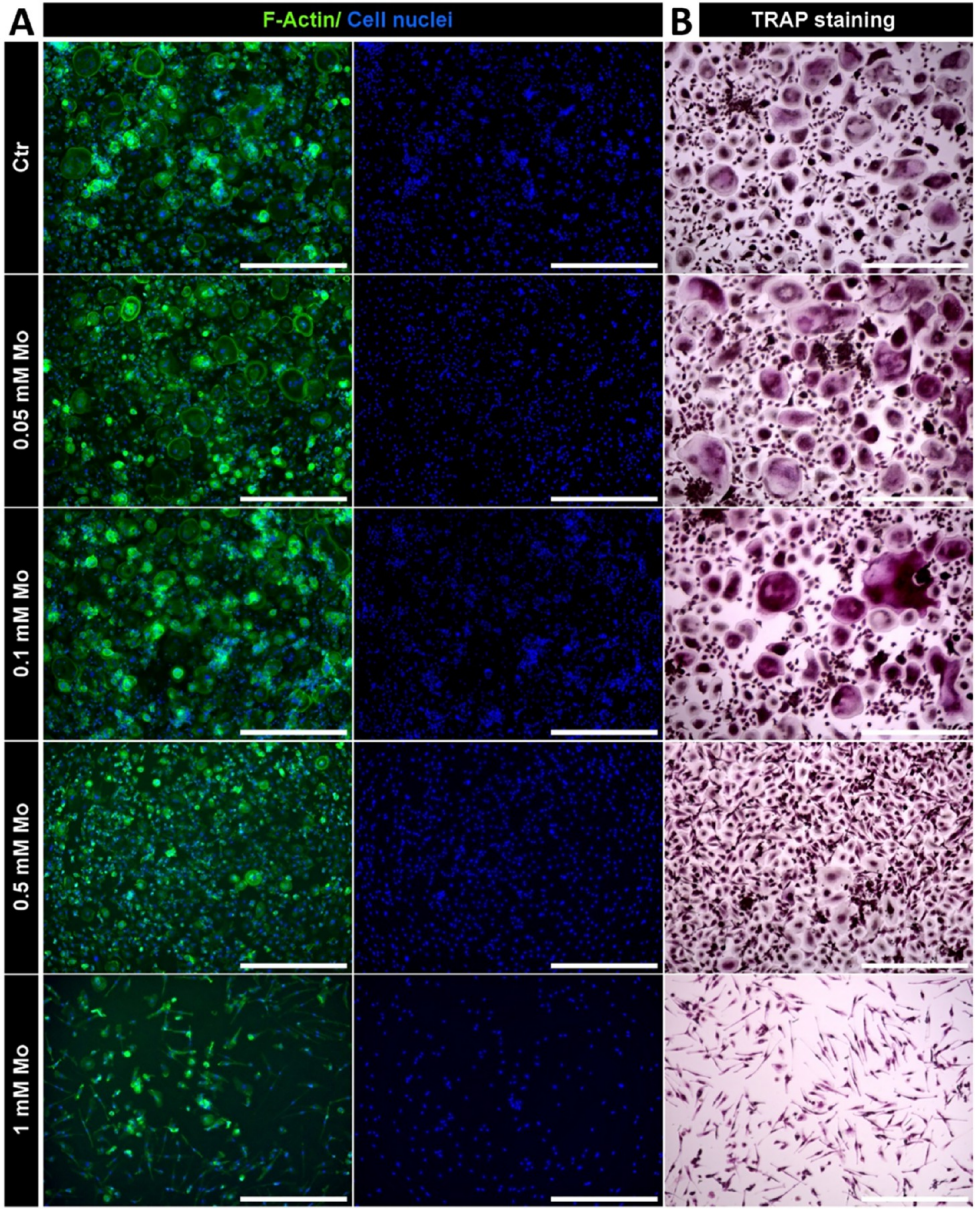
Impact of Mo extracts in different concentrations on RANKL- and MCSF-driven OC differentiation from PBMC in monoculture after 7 days treatment. (A) Fluorescence microscopic images. Cytoskeleton appears green (iFluor488 phalloidin) and cell nuclei appear blue (DAPI). (B) TRAP staining. Scale bars represent 500 μm.

### 3.3 Mo Induced Pro-Osteogenic and Pro-Angiogenic OB Markers as well as *VWF* of EC in Quadruple Culture

Since DNA content and morphology of OB were impaired at the highest Mo concentration (2 mM), this condition was excluded from further testing in quadruple culture. Effects on gene expression and ALP activity of OB in monoculture were strongest in the 1 mM group, thus 1 mM Mo was selected for testing in the bone quadruple culture.

HUVEC on top of OB in quadruple culture (Figure 5C) formed tube-like network structures with and without Mo (Figure 5A). However, network density of HUVEC was slightly lower in the Mo group compared to the Ctr. Separated CD31 channels visualizing HUVEC networks are presented in the Supporting Information (Figure S1). OB morphology was not impaired by Mo treatment. Alizarin red staining of transwell insert membranes with OB + HUVEC revealed intensified signals in the Mo-treated group (Figure 5B). Data on gene expression of OB + HUVEC and ALP activity are presented as summary data of four individual quadruple culture experiments. Statistics for the individual experiments, as well as ΔCt values of the control groups, are shown in the Supporting Information (Figure S2). Gene expression of OB markers *RUNX2* and *COL1A1* was downregulated in quadruple culture experiments in the presence of Mo compared to the Ctr (*p* < 0.0001; Figure 5D). No significant differences in *IBSP* gene expression were observed. In contrast, expression of *VEGFA*, *BGLAP*, *BMP-2*, RANKL (*TNFSF11*), and OPG (*TNFRSF11B*) was stimulated by Mo across the four experiments, with significant differences to the Mo-free Ctr (all *p* < 0.0001). Furthermore, *ALPL* gene expression and corresponding ALP activity of OB increased after Mo treatment (*p* < 0.0001) (Figure 5D,F). This is contrary to the decreased *ALPL* gene expression and activity in the presence of Mo in OB monoculture (Figure 3B,C). Moreover, RANKL/OPG ratio was increased in the presence of Mo in summary of the four individual experiments (Figure 5E).

**Figure 5:**
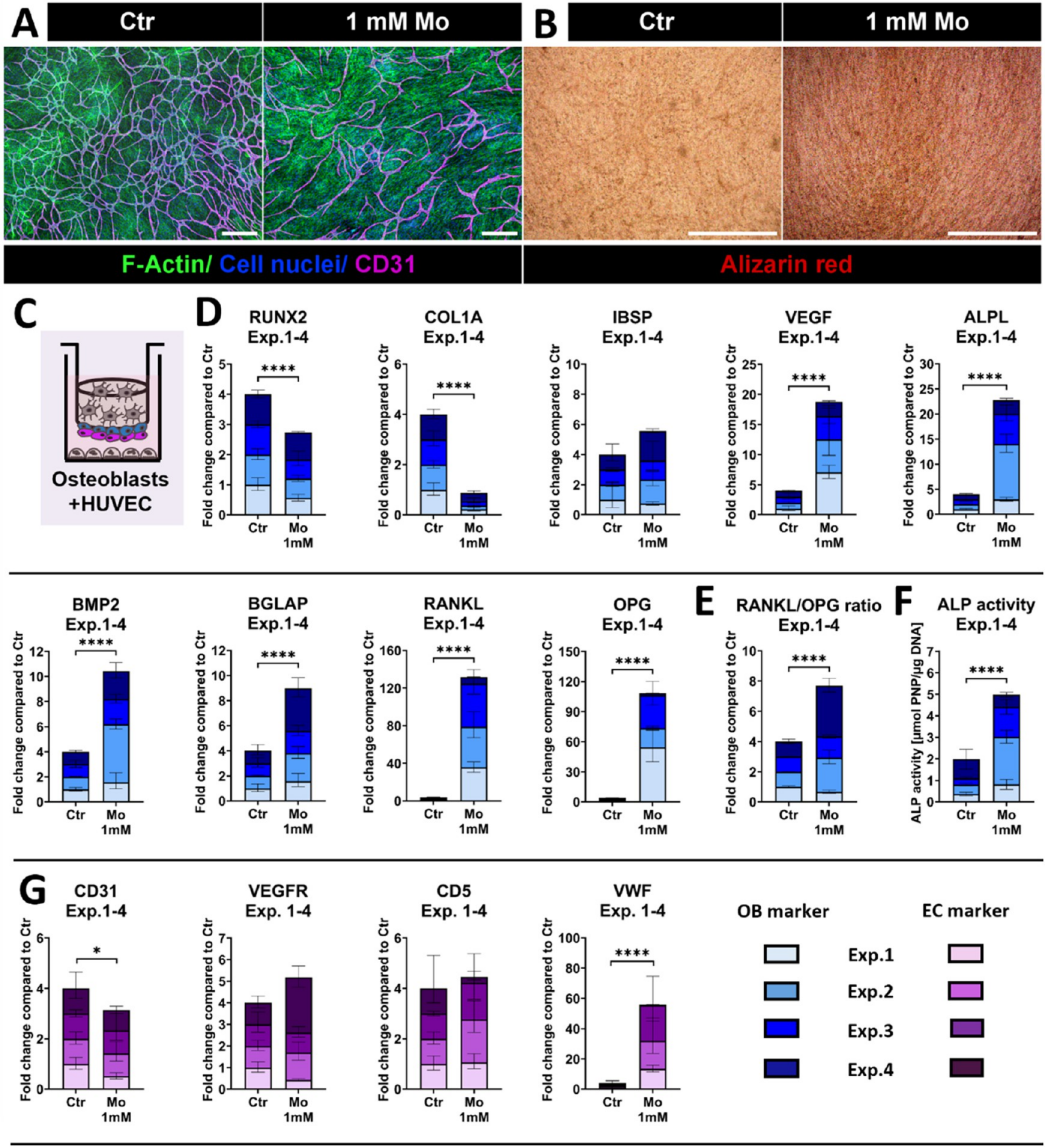
Impact of Mo on OB + HUVEC in summary of four individual quadruple culture experiments with distinct donor combinations after 14 days treatment. (A) Respective fluorescence microscopic images of OB + HUVEC in quadruple culture. The cytoskeleton is visualized in green (iFluor488 phalloidin), cell nuclei in blue (DAPI) and CD31 in magenta (Alexa Fluor 546). Scale bars represent 500 μm. (B) Alizarin red staining of OB + HUVEC in quadruple culture. Scale bars represent 500 μm. (C) Schematic illustration of OB + HUVEC in quadruple culture. (D) Gene expression of OB markers in quadruple culture, presented as fold change normalized to Ctr (summary data of four individual experiments; each *n* = 4 intradonor replicates). (E) RANKL/OPG ratio of OB in quadruple culture at gene expression level normalized to Ctr (summary data of four individual experiments, each *n* = 4 intradonor replicates). (F) ALP activity of OB in quadruple culture, normalized to Ctr (summary data of four individual experiments, each *n* = 3 intradonor replicates). (G) Gene expression of EC markers in quadruple culture presented as fold change normalized to Ctr (summary data of four individual experiments; each *n* = 4 intradonor replicates). *p < 0.05; **p < 0.01; ***p < 0.001; ****p < 0.0001.

Mo treatment also affected the expression of EC markers in quadruple culture (Figure 5G). CD31 (*PECAM1*) expression of HUVEC decreased in the presence of Mo, with significant difference compared to the Ctr (*p* < 0.05) across the four experiments. In contrast, *VWF* expression increased significantly after Mo treatment (*p* < 0.0001). Based on the summary of the four experiments, VEGFR (*KDR*) and *CDH5* expression were not significantly changed by Mo compared to the Ctr.

### 3.4 Mo Impaired OCY Differentiation in Quadruple Culture

OB embedded in collagen gel in transwell inserts differentiated into OCY in quadruple culture (Figure 6A,B). Three-dimensional OCY, characterized by multiple dendritic outgrowths and positive staining for DMP1, PDPN, and SOST, were observed in the Ctr group (Figure 6A). Mo treatment impaired OCY differentiation in quadruple culture. OCY were less dense, less branched, and more elongated in the presence of Mo. In addition, *PDPN* and OPG (*TNFRSF11B*) gene expression in OCY increased strongly with Mo across the four quadruple culture experiments (*p* < 0.0001) (Figure 6C). In contrast, *DMP1*, *MEPE*, *BGLAP*, and *SOST* gene expression decreased significantly after Mo treatment. The corresponding BGLAP and SOST protein concentrations in quadruple culture supernatants were reduced in the presence of Mo (*p* < 0.0001) (Figure 6E). SOST protein was detected in only one of the four experiments after Mo treatment. Furthermore, the RANKL/OPG ratio of OCY increased significantly in the presence of Mo, mainly due to the strong increase of OPG (*TNFRSF11B*) gene expression in three of the four experiments, along with reduced RANKL (*TNFSF11*) expression (Figure 6C,D). Statistics for the individual quadruple culture experiments, as well as ΔCt values of OCY in control groups, are shown in the Supporting Information (Figure S3).

**Figure 6:**
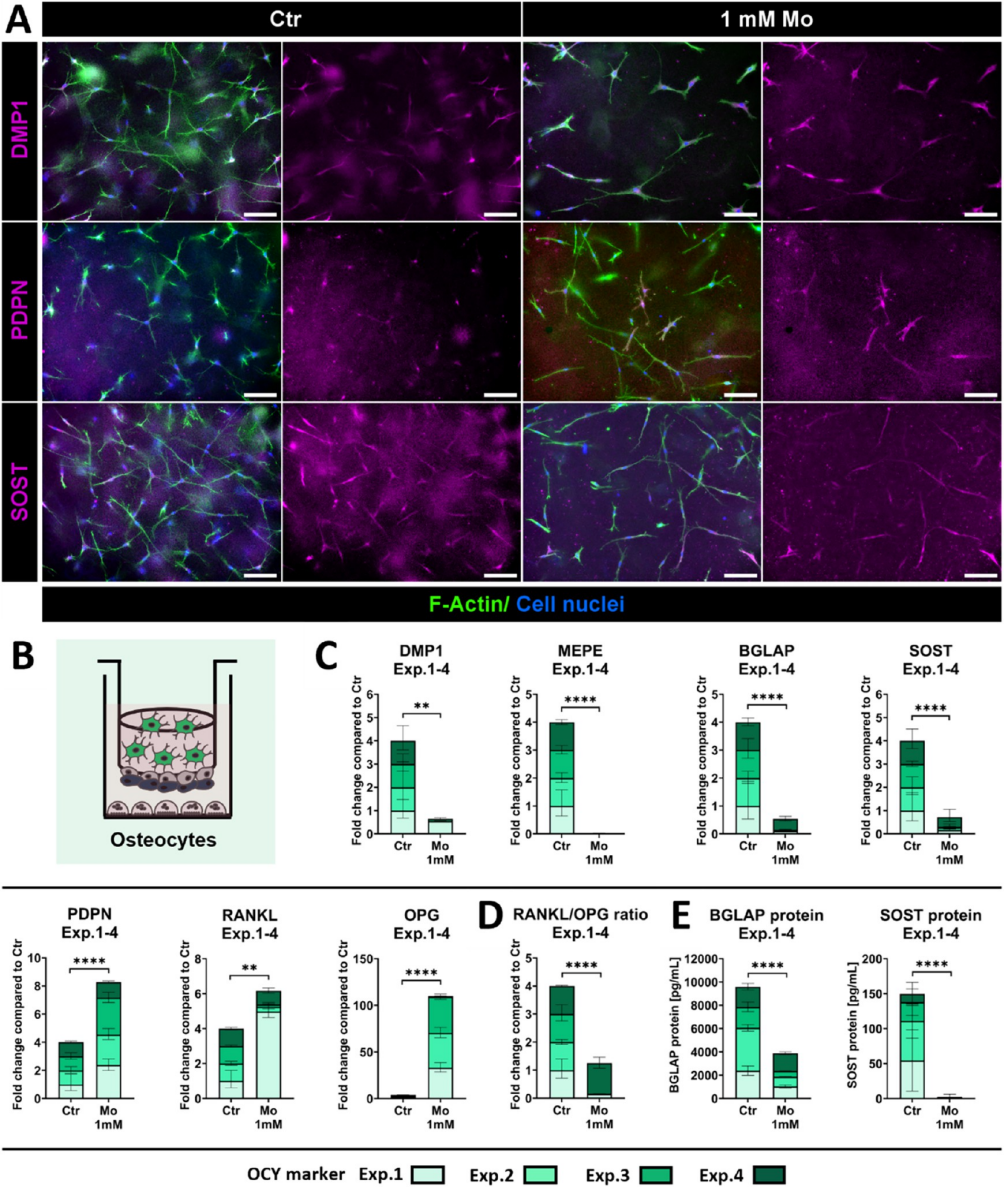
Impact of Mo on OCY in summary of four individual quadruple culture experiments with distinct donor combinations after 14 days treatment. (A) Respective fluorescence microscopic images of OCY in quadruple culture. The cytoskeleton is visualized in green (iFluor488 phalloidin), cell nuclei in blue (DAPI) and DMP1/PDPN/SOST in magenta (Alexa Fluor 546). Scale bars represent 100 μm. (B) Schematic illustration of OCY in quadruple culture. (C) Gene expression of OCY markers in quadruple culture presented as fold change normalized to Ctr (summary data of four individual experiments; each *n* = 4 intradonor replicates). (D) RANKL/OPG ratio of OCY in quadruple culture at gene expression level (summary data of four individual experiments; each *n* = 4 intradonor replicates). (E) BGLAP and SOST protein concentrations in quadruple culture supernatants (summary data of four individual experiments; each *n* = 3 intradonor replicates). n.d. (not detectable). **p < 0.01; ****p < 0.0001.

### 3.5 Mo Inhibited OC Formation in Quadruple Culture

Under Ctr conditions, PBMC in quadruple culture differentiated into rounded, multinucleated OC (Figure 7A,B). The presence of Mo inhibited OC formation, as indicated by the accumulation of multiple mononuclear cells, which were significantly smaller than the OC in the Ctr group. While *CA2* gene expression was not impaired, *ACP5* and *CTSK* gene expression decreased significantly after Mo treatment in all independent quadruple culture experiments (across Exp.1–4 *p* < 0.0001) (Figures 7C and S4). Consistent with the gene expression data, the corresponding OC-specific TRAP and CTSK activities were strongly reduced after Mo treatment in quadruple culture (Figure 7D). Statistics for the individual quadruple culture experiments, as well as ΔCt values of OC in control groups, are shown in the Supporting Information (Figure S4).

**Figure 7:**
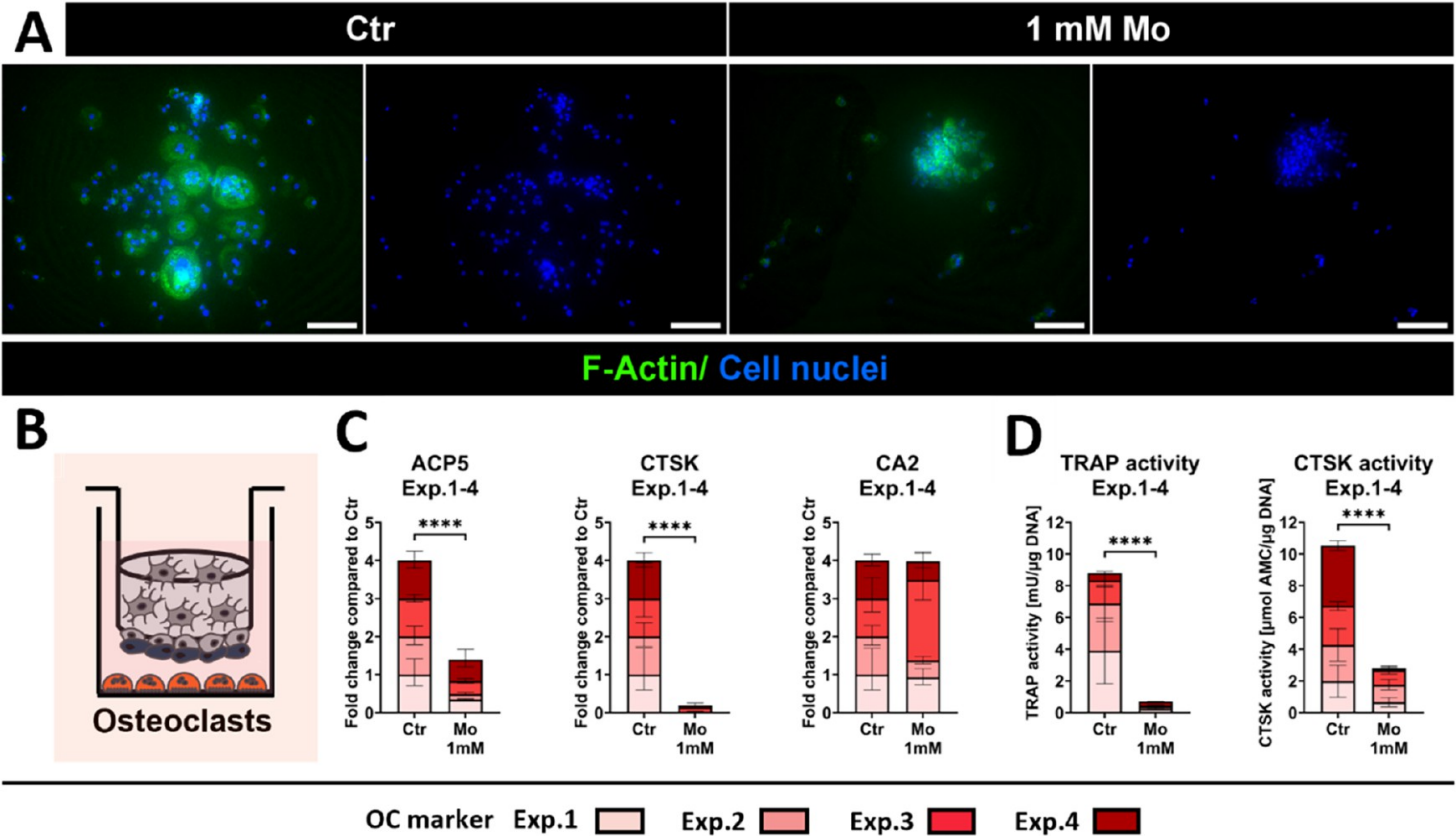
Impact of Mo on OC in summary of four individual quadruple culture experiments with distinct donor combinations after 14 days treatment. (A) Respective fluorescence microscopic images of OC in quadruple culture. The cytoskeleton is visualized in green (iFluor488 phalloidin), and cell nuclei in blue (DAPI). Scale bars represent 100 μm. (B) Schematic illustration of OC in quadruple culture. (C) Gene expression of OC markers in quadruple culture presented as fold change normalized to Ctr (summary data of four individual experiments; each *n* = 4 intradonor replicates). (D) OC specific enzyme activities in quadruple culture (summary data of four individual experiments; each *n* = 3 intradonor replicates). ****p < 0.0001.

### 3.6 Mo Induced Pro-Inflammatory TNFα

In addition to cell-type-specific analyses, secretion of the proinflammatory proteins tumor necrosis factor α (TNFα) and interleukin-1β (IL-1β) was quantified in cell culture supernatants as an outcome of the whole quadruple culture. TNFα-concentration increased significantly in the presence of the Mo extract compared to the control groups across the four individual experiments (*p* < 0.0001) (Figure 8A). In contrast, IL-1β protein was not detectable in quadruple culture supernatants, regardless of Mo extract supplementation (Figure 8B).

**Figure 8:**
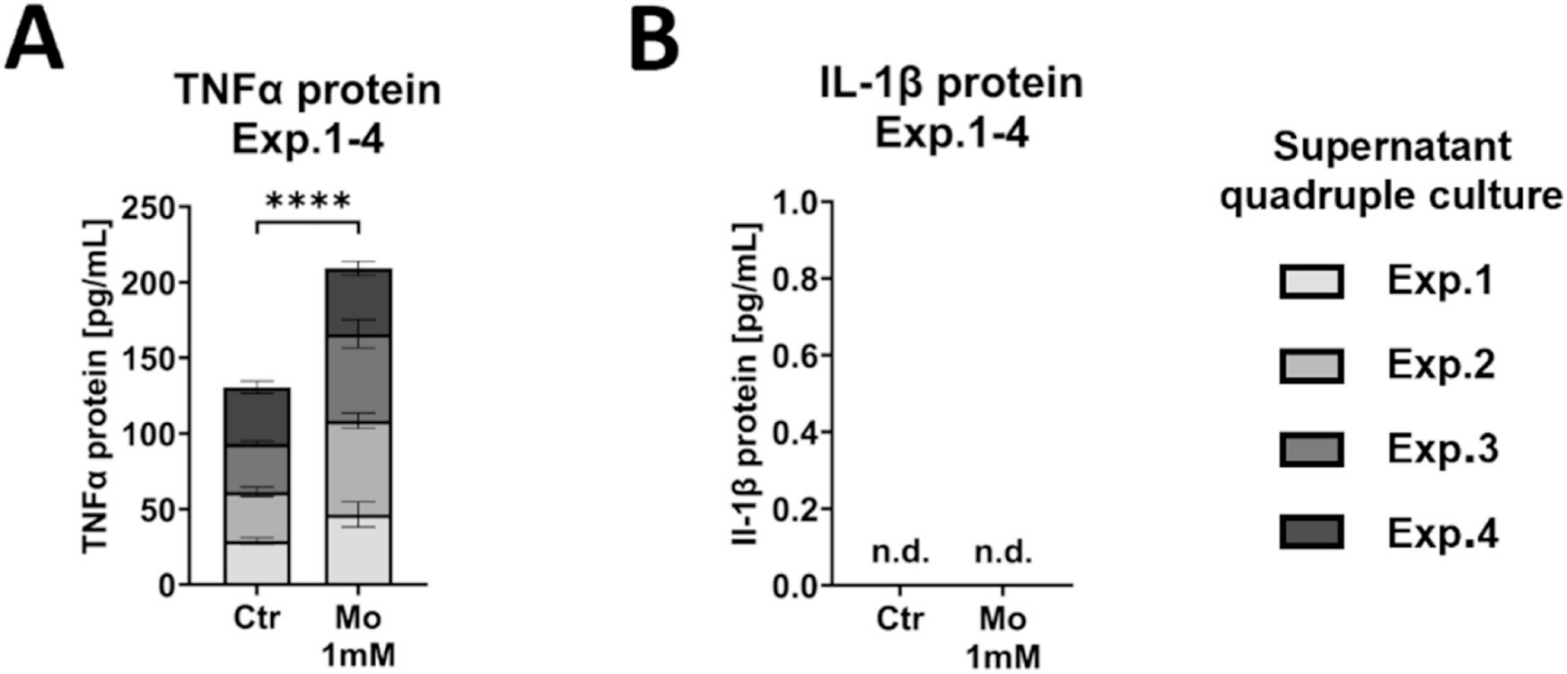
Impact of Mo on pro-inflammatory protein secretion in supernatants of four individual quadruple culture experiments with different donor combinations after 14 days treatment. (A) TNFα protein concentrations (summary data of four individual experiments; each *n* = 3 intradonor replicates). (B) IL-1β protein concentrations (summary data of four individual experiments; each *n* = 3 intradonor replicates). n.d. not detectable. **** *p* < 0.0001.

## 4 Discussion

The increasing prevalence of bone-related diseases and the desire to improve patient outcomes drive the search for new bone replacement materials. Given the limitations of current bone substitutes, such as insufficient bioavailability, biocompatibility, biodegradability, and mechanical properties, as well as inadequate osteointegration, there is a need to investigate alternative options overcoming these challenges. In particular, there is a demand for moldable, patient-specific and resorbable implants supporting bone regeneration. In this context, metallic Mo is recently discussed as a candidate to fulfill these criteria, and there is growing interest in its potential applications. However, systematic investigations addressing its effects within a multicellular human bone environment remain limited. Therefore, the present study evaluated the biological impact of Mo using a recently developed in vitro bone model as quadruple culture with primary human OB, OCY, OC, and HUVEC. This multicellular system allows for cellular crosstalk relevant to bone remodeling and enables simultaneous assessment of osteogenesis, osteocytogenesis, osteoclastogenesis, and endothelial network formation within a single experimental system, as shown previously on morphological, gene expression, enzyme activity, and protein secretion levels.^16^ In contrast, to the emerging organoid technology, which relies on self-organization to develop heterogeneous and partially controllable 3D architectures, the quadruple culture model is based on a compartmentalized transwell approach. Although organoids are well suited to studying tissue morphogenesis and spatial organization by mimicking the native hierarchical structure, their differentiation from stem cells includes structural variability, which limits experimental control, reproducibility, and standardization.^34,35^ In addition, generating bone organoids that reliably contain all three main bone cell types and EC remains technically challenging. Conversely, the predefined spatial arrangement and controlled cellular composition of quadruple cultures enable the targeted investigation of specific signaling pathways and cell-type-resolved analyses while preserving crosstalk. This higher degree of experimental control and cellular composition makes the quadruple culture particularly advantageous for mechanistic and cell type-specific studies of bone remodeling in a multicellular system.

Instead of solid Mo specimens, a Mo powder extract was applied for technical reasons. This approach ensured uniform exposure of all cell types in the quadruple culture and allowed investigation of degradation-related soluble species. However, extract-based approaches do not reproduce local concentration gradients, surface-mediated interactions, corrosion layer formation, or mechanical loading conditions that would occur in vivo. Accordingly, the present findings should be interpreted as mechanistic insights into cellular response to soluble Mo species rather than as direct prediction of implant performance. Nevertheless, these fundamental insights are essential for biomaterial development.

Incubation of Mo powder in PBS resulted in high Mo concentrations (∼50 mM), attributed to the high surface-to-liquid ratio of the powder. However, such concentrations are unlikely to be present in vivo, where the effective surface area relative to surrounding tissue fluid is considerably lower. Therefore, extracts were diluted for quadruple culture experiments. Previous initial in vivo studies demonstrated gradual degradation of Mo implants in the range of 13.5 μm/year after implantation into the abdominal aorta of Wistar rats.^22^ However, quantitative in vivo degradation rates of Mo within mineralized bone tissue are currently insufficiently characterized and require further in vivo investigations. Nevertheless, for understanding the fundamental cellular processes induced by biodegradable Mo on bone turnover, the application of a Mo extract is significantly meaningful to provide solid evidence from in vitro studies necessary for moving on to in vivo and clinical studies during biomaterial development.

Mo extracts exhibited biocompatibility with OB up to 1 mM Mo. Furthermore, OB DNA content increased up to this concentration, suggesting enhanced proliferation and/or survival (Figure 2A,B). These findings are consistent with previous in vitro viability assessment of human bone marrow-derived mesenchymal stem cells treated with Mo trioxide: increased viability with concentrations between 0.5 and 3 mM and reduced viability with 4–10 mM.^36^ In addition, undiluted Mo extracts prepared similarly to those tested in the present quadruple culture displayed no impaired viability of L2929 mouse fibroblast cells.^23^ However, the exact concentrations of Mo in the extracts were not provided. Viability of PBMC and OC was also not impaired by Mo extracts in the present study with concentrations up to 1 mM and 0.1 mM, respectively. Previous in vivo studies confirmed the excellent biocompatibility of Mo observed in vitro, further demonstrating the stability and degradation of Mo implants. Hoppe et al. implanted metallic Mo in domestic pigs and observed no adverse tissue reactions but early signs of degradation after 54 days.^24^ Additionally, Mo implants in the nuchal folds of Wistar rats did not cause significantly increased inflammatory reaction compared to titanium implants.^21^ However, degradation of Mo implants was minimal, likely due to the formation of capsules, which has been observed with other implants. In another study, Mo wires were implanted into the abdominal aorta of Wistar rats, and uniform degradation without localized corrosion was observed. Degradation of the wires did not induce increased Mo levels in serum, urine, liver, or kidneys, suggesting physiological transport and excretion of dissolved Mo from degrading implants.^22^

Since Mo fulfills the fundamental criteria for a resorbable bone replacement material–namely, biocompatibility and biodegradability–its biological effects on bone cells came into the focus of recent bone research. The bioavailable form of Mo is the molybdate anion (MoO_4_^2–^).^20^ Therefore, observed changes in cellular behavior of OB, OCY, OC and HUVEC in quadruple culture treated with Mo extracts may be caused by the presence of molybdate anions. After incubating the Mo powder in PBS (1 g/mL), a dark blue extract containing ∼50 mM Mo was obtained. The characteristic blue color indicated the formation of Mo blue complexes. In the presence of phosphate, phosphomolybdenum blue species may have formed following partial reduction of polymolybdates.^37^ Interestingly, the blue color of Mo extracts was only observed in PBS. After dilution in cell culture medium for quadruple cultures containing FCS and other supplements, the medium initially exhibited a bluish color. However, after incubating at 37 °C already after 24 h, the additional bluish color vanished (Figure S5). We conclude that the intensely colored Mo blue complexes were unstable in the culture medium and dissolved, explaining the loss of color. The presence of proteins and other biomolecules in the quadruple culture medium likely served as redox partners that oxidized the Mo blue complexes, resulting in bioavailable molybdate anions. The stable bluish color of PBS-diluted Mo extracts after 24 h at 37 °C in an incubator indicates that temperature, pH, and CO_2_ levels in the cell culture medium do not affect the stability of Mo blue complexes (Figure S5).

Regarding OB, the presence of Mo extracts induced osteogenic differentiation in quadruple culture, including *ALPL* gene expression and ALP activity, as well as *BGLAP* and *BMP2* gene expression, accompanied by enhanced mineral deposition (Figure 5). Notably, the pro-osteogenic response in quadruple culture stands in contrast to reduced ALP activity observed in OB monocultures treated with Mo extracts (Figure 3). This finding underscores the critical importance of multicellular models over monoculture approaches. In monoculture, OB respond to Mo in isolation, whereas in quadruple culture, paracrine signals from OCY, OC, and HUVEC can modulate the OB response. EC-derived factors have been shown to stimulate osteogenic differentiation,^6,9^ and OCY-derived signals contribute to OB regulation as well. The presence of these intercellular signaling axes in the quadruple culture likely accounts for the enhanced osteogenic response. Future studies, focusing particularly on Mo-induced pro-osteogenic responses are needed to investigate involved signaling pathways in more detail (by applying specific inhibitors in vitro). However, this is beyond the scope of the present quadruple culture study, which focuses on the resulting output in the presence of all four cell types. The osteogenic potential of Mo has been reported in previous studies. More specifically, Mo-doped bioactive glass nanoparticles enhanced ALP activity and integrin binding sialoprotein gene expression, as well as biomineralization of adipose-derived stem cells.^27^ Furthermore, treatment of bone marrow-derived stromal cells with Mo trioxide induced ALP activity, *BGLAP* gene expression, and ECM formation,^36,38^ which was similarly observed in the present study with intensified alizarin red staining after Mo treatment. Additionally, stimulation of the osteogenic markers ALP and BMP-2, as seen in quadruple cultures with Mo extracts, was observed in human bone mesenchymal stem cells cultivated on ceramic scaffolds containing Mo-doped bioactive glasses^39^ and in MC3T3-E1 cells cultivated in a Mo-containing hydrogel based on GelMa.^40^ The authors concluded that these effects result from direct scavenging of ROS and activation of the PI3K/Akt signaling pathway by Mo. In addition, the JAK/STAT3 signaling pathway is suggested to be involved in Mo-mediated activation of osteogenic differentiation and mineralization, as recently demonstrated in periodontal ligament stem cells following Mo treatment.^41^ These proposed signaling mechanisms remain to be experimentally validated in the present quadruple culture study, for example, through inhibitor studies. In contrast to the positive influence of Mo on *ALPL*, *BGLAP*, and *BMP2* gene expression, *COL1A1* gene expression of OB decreased notably in Mo-supplemented quadruple cultures (Figure 5). These findings align with significantly reduced *COL1A1* expression in bone marrow-derived mesenchymal stem cells treated with Mo trioxide.^36^ As type I collagen is the most abundant protein in bone tissue, it is not yet clear whether Mo is able to promote new bone formation in vivo. In addition, reduced COL1A could lead to compromised matrix quality or mechanical integrity. Therefore, further in vivo studies are essential to clarify the predominant effect of Mo on bone regeneration. One possible explanation is that Mo accelerates maturation toward a mineralization-oriented phenotype, resulting in temporally shifted *COL1A1* expression.

Besides pro-osteogenic effects, Mo treatment also modulated angiogenic parameters, as *VEGFA* expression of OB was increased in quadruple cultures (Figures 3 and 5). This suggests activation of pro-angiogenic signaling pathways that may support early bone regeneration. Previous reports have shown that the presence of molybdate ions triggers the activation of the HIF1α signaling pathway,^39^ which is suggested to have caused the observed *VEGFA* induction in quadruple cultures. Although HUVEC retained the ability to form network-like structures, a reduction in CD31 *(PECAM1)* expression and network density was observed (Figure 5). However, no cytotoxic effects of Mo on HUVEC were detected at concentrations up to 1 mM (Figure 2C). These findings indicate a mixed angiogenic response of Mo in vitro and further in vivo validation is necessary to clarify the angiogenic responses. In addition, there are partially conflicting findings in previous studies regarding Mo’s impact on angiogenesis, likely reflecting differences in chemical form, concentration, and experimental design.^42^ While Mo trioxide nanoparticles inhibited EC migration and exhibited anti-angiogenic effects in a chorioallantoic membrane (CAM) and chick aortic ring assay,^43^ Khandia et al. reported on pro-angiogenic effects of Mo trioxide in the CAM assay but reduced blood vessel density after sodium molybdate (Na_2_MoO_4_·2H_2_O) treatment.^44^ In addition, Moll et al. confirmed rather anti-angiogenic properties of Mo as a part of bioactive glasses in vitro and in ovo.^45^ Nevertheless, Mo extracts did not inhibit the formation of tube-like structures by HUVEC in bone quadruple cultures, and VEGF as major player in regulation of angiogenesis, but also in the context of osteogenic differentiation, might be involved in the observed upregulation of osteogenic gene expression.^46^ Moreover, the presence of OB and OCY in quadruple cultures may have protected HUVEC from Mo-induced anti-angiogenic effects. To our knowledge, this is the first study investigating the impact of Mo in a multicellular in vitro environment allowing the crosstalk between OB, OCY, OC and HUVEC. Mo extracts strongly induced gene expression of RANKL (*TNFSF11*) and OPG (*TNFRSF11B*) in OB and OCY in quadruple cultures (Figures 5 and 6). However, specifically in OCY, RANKL/OPG ratio shifted toward OPG dominance, which positively regulates microvessel formation, while RANKL is an angiogenic inhibitor.^47^ In addition, we observed for the first time a strong increase of *VWF* expression in HUVEC after Mo treatment in quadruple cultures. VWF plays a critical role in blood clotting by facilitating platelet adhesion and aggregation at sites of vascular injury. VWF deficiency or dysfunction leads to bleeding problems, while excessive VWF activity can cause thrombotic events.^48^ In addition, the induction of VWF hints at the activation of inflammatory pathways by Mo, as VWF is involved in leukocyte recruitment.^49,50^ This suggestion is supported by the observed induction of the pro-inflammatory protein TNFα in quadruple culture supernatants (Figure 8). A macrophage-related inflammatory response of RAW 264.7 cells to degradation products of biodegradable Mo implants, indicated by induced secretion of pro-inflammatory cytokine interleukin 6, was also reported before.^51^ In the context of bone healing and regeneration, the induction of inflammatory pathways, especially when coupled with osteogenic pathways, can be beneficial because the initial phase of bone healing is an inflammatory phase, facilitating the removal of debris from damaged tissue by immune cells, as well as the recruitment of cells and signals inducing bone formation.^52^ However, TNFα is a pleiotropic cytokine with concentration- and context-dependent effects. While low-level, transient TNFα secretion supports osteogenic differentiation and angiogenesis during early stages of healing,^53^ sustained elevated TNFα can inhibit OB differentiation, promote osteoclastogenesis, and impair bone quality.^54^ The absence of detectable IL-1β in the present study suggests a selective, rather than broadly pro-inflammatory response. Nevertheless, TNFα signaling which is potentially beneficial in early healing but harmful if prolonged, warrants investigation in longer-term and in vivo studies.

On the side of OC, 1 mM Mo directly inhibited OC differentiation from PBMC in monoculture, without compromising precursor viability (Figures 2C and 4). Therefore, Mo directly interfered with OC formation in the presence of RANKL and MCSF in PBMC monocultures. In addition, treatment of bone quadruple cultures provided insights into indirect effects of Mo on OC differentiation through modulation of OB and OCY behavior, as these cell types are the main source for RANKL and OPG in bone tissue. Observed induction of OPG (*TNFRSF11B*) and RANKL (*TNFSF11*) gene expression in OB and OCY after Mo treatment significantly shifted the RANKL/OPG ratio toward OPG and therefore toward an anti-osteoclastic profile in OCY (Figure 6). Given that OCY are the most abundant cell type in bone tissue and recognized as the dominant regulators of osteoclastogenesis in vivo,^55,56^ we conclude that the OCY-mediated anti-osteoclastic signal predominates. This suggests a dual inhibition mechanism of OC differentiation in quadruple cultures in the presence of Mo extracts comprising direct interference with OC differentiation and indirect modulation via paracrine signaling. Consequently, OC-specific gene expression and enzyme activities were strongly reduced in the Mo groups (Figure 7). Formation of multinucleated OC is mainly driven by the RANK/RANKL/OPG system. While RANKL induces the fusion of mononuclear OC progenitors into OC, OPG blocks this mechanism.^57^ The stimulation of OPG (*TNFRSF11B*) gene expression and OPG protein concentration in cell culture supernatant of bone marrow-derived stem cells treated with Mo^36,45^ and inhibition of OC formation from murine progenitors in the presence of Mo^26^ previously evidenced the anti-osteoclastic effect of Mo. Furthermore, the formation of OC is highly energy demanding, and during phenotypic changes from mononuclear progenitors to multinucleated OC the number and size of mitochondria increases drastically.^58–60^ Since Mo ions can accumulate in mitochondria, influencing their function, the inhibitory effect of Mo on osteoclastogenesis may in part be related to its effects on mitochondrial biogenesis.^61^ This hypothesis is supported by the previously reported decrease in mitochondrial ROS production in OC in the presence of Mo ions.^26^ Simple cytotoxic effects of Mo extracts on PBMC in quadruple cultures can be excluded, since Mo extracts with a concentration of up to 2 mM Mo did not reduce viability of both PBMC and mature OC below 75% compared to a Mo-free control (Figure 2C,D).

So far, to our knowledge, no studies were conducted on Mo’s effect on primary human OCY. Kanaji et al. treated MLO-Y4 OCY-like cells with cobalt-chromium-Mo alloy particles. However, pure Mo was not tested, therefore conclusions about Mo’s impact on the observed induced inflammatory responses cannot be drawn.^62^ Furthermore, MoCl_5_ did not cause cytotoxic effects on MLO-Y4 cells up to 0.5 mM.^63^ Therefore, the present bone quadruple culture study investigated the impact of soluble Mo species on primary human OCY for the first time. Mo exposure strongly reduced expression of late OCY marker genes, especially *DMP1*, *MEPE*, and *SOST*. Additionally, the OCY phenotype was impaired by reduced formation of sprouted dendritic outgrowths and the presence of a more elongated cell morphology (Figure 6). Therefore, we conclude that Mo decreased OCY differentiation. Solely *PDPN*, RANKL (*TNFSF11*), and OPG (*TNFRSF11B*) were upregulated in OCY in Mo extract groups. PDPN is one of the earliest OCY markers involved in dendrite formation.^64^ The upregulation of *PDPN*, alongside the reduced expression of late OCY markers and insufficient dendrite formation in quadruple cultures treated with Mo extracts suggests delayed OCY maturation toward early OCY stages rather than a complete inhibition of differentiation. Importantly, the late OCY marker SOST is an inhibitor of canonical WNT signaling.^65^ Reduced SOST may consequently permit enhanced WNT pathway activity, which is known to promote osteogenesis and mineralization. Therefore, the maintenance of an early OCY state induced by Mo could contribute indirectly to sustained osteogenic activity. From a regenerative perspective, OCY maturation does not limit initial phases of bone healing, which are dominated by inflammatory processes, vascularization, and OB activity. Since Mo extracts promoted osteogenic differentiation, mineralization, and angiogenic signaling in quadruple cultures, a delay in OCY maturation is unlikely to compromise early bone formation. However, given the central role of mature OCY in mechanotransduction, long-term bone quality regulation, and remodeling, consequences of altered osteocytogenesis must be evaluated in vivo. It remains to be determined whether OCY maturation recovers or if prolonged Mo exposure results in persistent impairment of the OCY network, which leads to impaired bone quality.

Overall, the results showed mostly consistent directionality of Mo-induced effects across the four quadruple culture experiments with different donor combinations (Figures S2–S4). This supports the robustness of the observed effects independent of donor-specific bias because at least cells from two different donors were used for all cell types within the four quadruple culture experiments. However, when using primary cells, variations among donors could result from multiple factors, such as age, gender, pre-existing diseases, and lifestyle. Therefore, to avoid donor-specific bias, distinct donors should be used for each cell type. A limitation of the present quadruple culture study is the single end point (14 days) analysis, which captures differentiation responses but does not provide information on temporal dynamics of the observed effects. Including earlier and later time points would be particularly interesting for inflammatory and angiogenic responses, as well as effects on mineralization. Therefore, analyzing distinct end points is an important direction for future studies, which are beyond the scope of the present study due to the resource- and time-intensive nature of the quadruple culture model. Furthermore, future studies should include a broader range of Mo concentrations (μM range) to establish dose–response relationships in quadruple culture and better approximate potential physiological exposure levels in vivo. In addition, validation of the present findings using solid Mo specimens in dynamic degradation systems would be an important step toward translational relevance, as well as toward in vivo studies using solid Mo samples as bone graft material.

In summary, exposure to Mo extracts in the bone quadruple culture induced a coordinated biological response providing new evidence for the applicability of Mo as bone replacement material promoting bone healing. While extract-based findings cannot directly predict the behavior of metallic implants, the applied multicellular in vitro model allowed mechanistic insights into cell type specific reactions relevant to biodegradable Mo-based materials. Thus, the applied bone model is suitable for initial in vitro screening of biomaterial candidates, enabling cell type-specific and intercellular responses with exclusively primary human cells while reducing the need and reliance on costly and ethically questionable animal testing. In addition to pure metallic implants, Mo could be incorporated into existing biomaterials, such as bioactive glasses or calcium phosphate cements, as already published in some initial studies.^26,45^ Calcium phosphate cements (CPCs) are already used in orthopedic applications as biocompatible, osteoinductive bone substitutes. However, their mechanical properties, particularly toughness and brittleness, limit their applicability in load-bearing areas.^66,67^ Modifying CPCs with Mo could be a strategy to improve their mechanical and osteoconductive properties, contributing to improvements in growth factor-free bone tissue engineering.

## 5 Conclusion

The presence of Mo extracts in human bone quadruple cultures modulated bone cell dynamics in a multifunctional manner. Osteogenic differentiation and mineralization of OB were induced, while OC formation was inhibited. Concurrent induction of VEGF indicated activation of angiogenic signaling, although a simultaneous reduction in CD31 (*PECAM1*) expression and network density suggests a complex, mixed endothelial response that requires further characterization. The combined pro-osteogenic, angiogenic, and anti-osteoclastogenic effects of Mo extracts observed in the bone quadruple culture, in addition to excellent biocompatibility with all cell types assessed in respective monocultures, gave new mechanistic evidence for the potential of Mo as a biodegradable bone replacement material (Figure 9). In addition, this is the first study providing systematic investigations of Mo’s impact on crosstalk between primary human OB, OCY, OC, and HUVEC, offering mechanistic insight into its influence on bone cell differentiation and interaction. Together, these findings establish a cellular and biological foundation for further research on the rational design of Mo-containing biomaterials aimed at promoting bone regeneration, and the extract-based nature of the present study should be considered when translating these findings toward implant-relevant settings.

**Figure 9:**
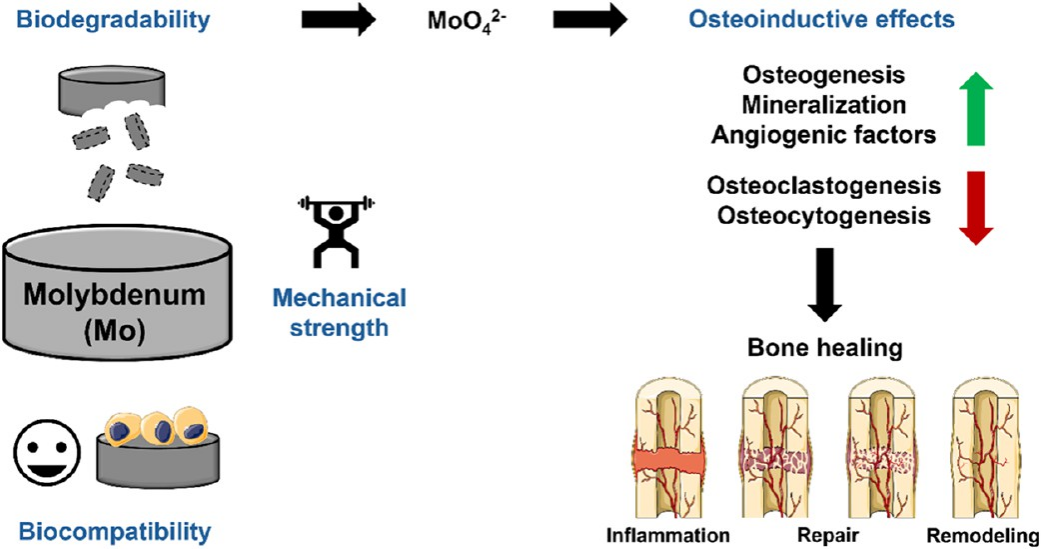
Schematic representation of Mo’s properties supporting its use as a biomaterial for bone regeneration, as well as cellular effects induced by Mo extracts in bone quadruple cultures. This figure includes adapted illustrations from Servier Medical Art (Servier; https://smart.servier.com/), licensed under a Creative Commons Attribution 4.0 unported license.

## Supplementary Material



## Data Availability

All data reported within this article are available in Supporting Files or will be made available upon request.

## Notes

Fluorescence microscopic images of OB + HUVEC with separated CD31 channel (Figure S1), gene expression data of individual experiments including ΔCt values of the control groups (Figures S2–S4), photographs of Mo extracts diluted in medium or PBS (Figure S5) (PDF)
