# Supplementary material for: Advancing Biomaterials Evaluation: A Human Quadruple Bone Cell Culture Reveals Molybdenum-Driven Pro-Osteogenic and Anti-osteoclastogenic Responses
Source: ACS Appl Mater Interfaces. 2026 May 7;18(19):27314–31. doi: 10.1021/acsami.6c04674 (PMC13195570; doi:10.1021/acsami.6c04674)
Supplement: Supplementary file 1 [file am6c04674_si_001.pdf]

Supporting Information

Advancing biomaterials evaluation: a human quadruple bone cell culture reveals molybdenum-driven pro-osteogenic and anti-osteoclastogenic responses

Katharina Wirsig[a], Anne Bernhardt[a]*

[a] Centre for Translational Bone, Joint- and Soft Tissue Research, Faculty of Medicine and University Hospital Carl Gustav Carus, TUD University of Technology, Fetscherstraße 74, 01307 Dresden, Germany

* Corresponding author: Anne.bernhardt@tu-dresden.de

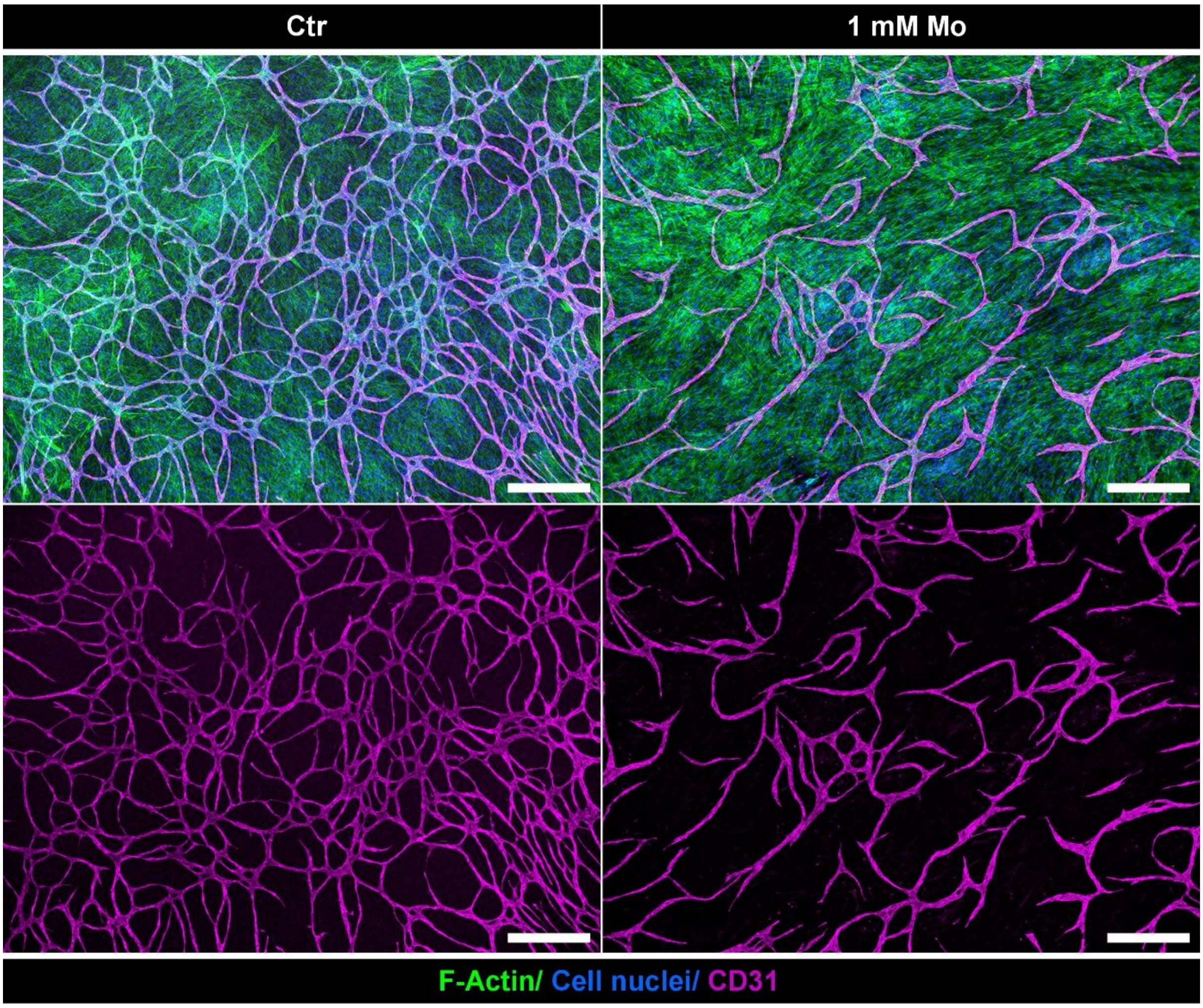

Figure S1: Fluorescence microscopic images of OB+HUVEC in quadruple culture after 14 days treatment. Cytoskeleton appears green (iFluor488 phalloidin), cell nuclei appear blue (DAPI) and CD31 appears magenta (Alexa Fluor 546). Scale bars represent 500 µm.

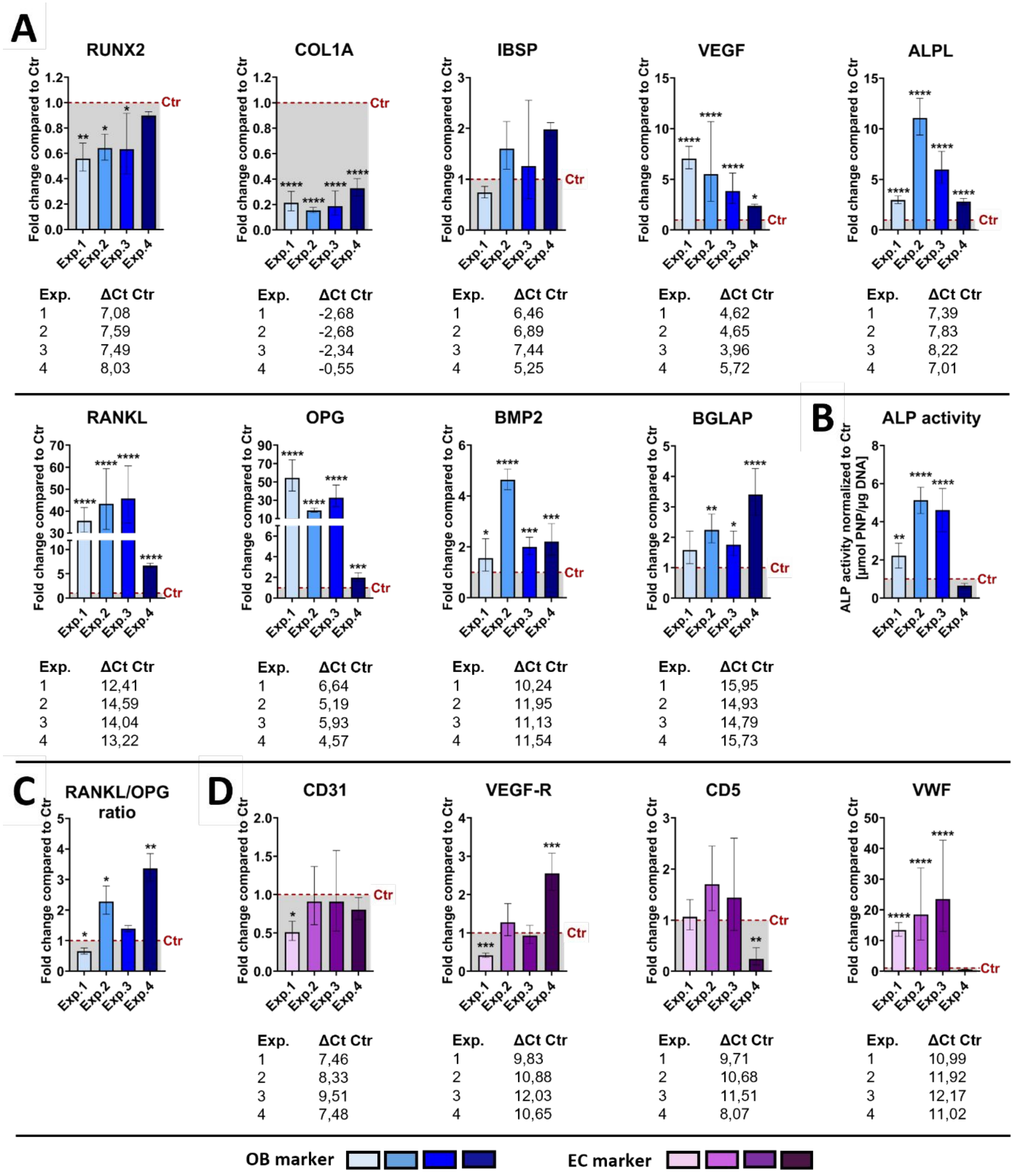

Figure S2: Impact of Mo on OB + HUVEC in four individual quadruple cultures experiments with different donor combinations after 14 days treatment. (A) Gene expression of OB markers presented as fold change normalized to Ctr (each n = 4), as well as ΔCt values in control groups. (B) ALP activity of OB normalized to Ctr (each n = 3). (C) RANKL/OPG ratio of OB on gene expression level normalized to Ctr (each n = 4). (D) Gene expression of EC markers presented as fold change normalized to Ctr (each n = 4), as well as ΔCt values in control groups. *$p < 0.05$; **$p < 0.01$; ***$p < 0.001$; ****$p < 0.0001$.

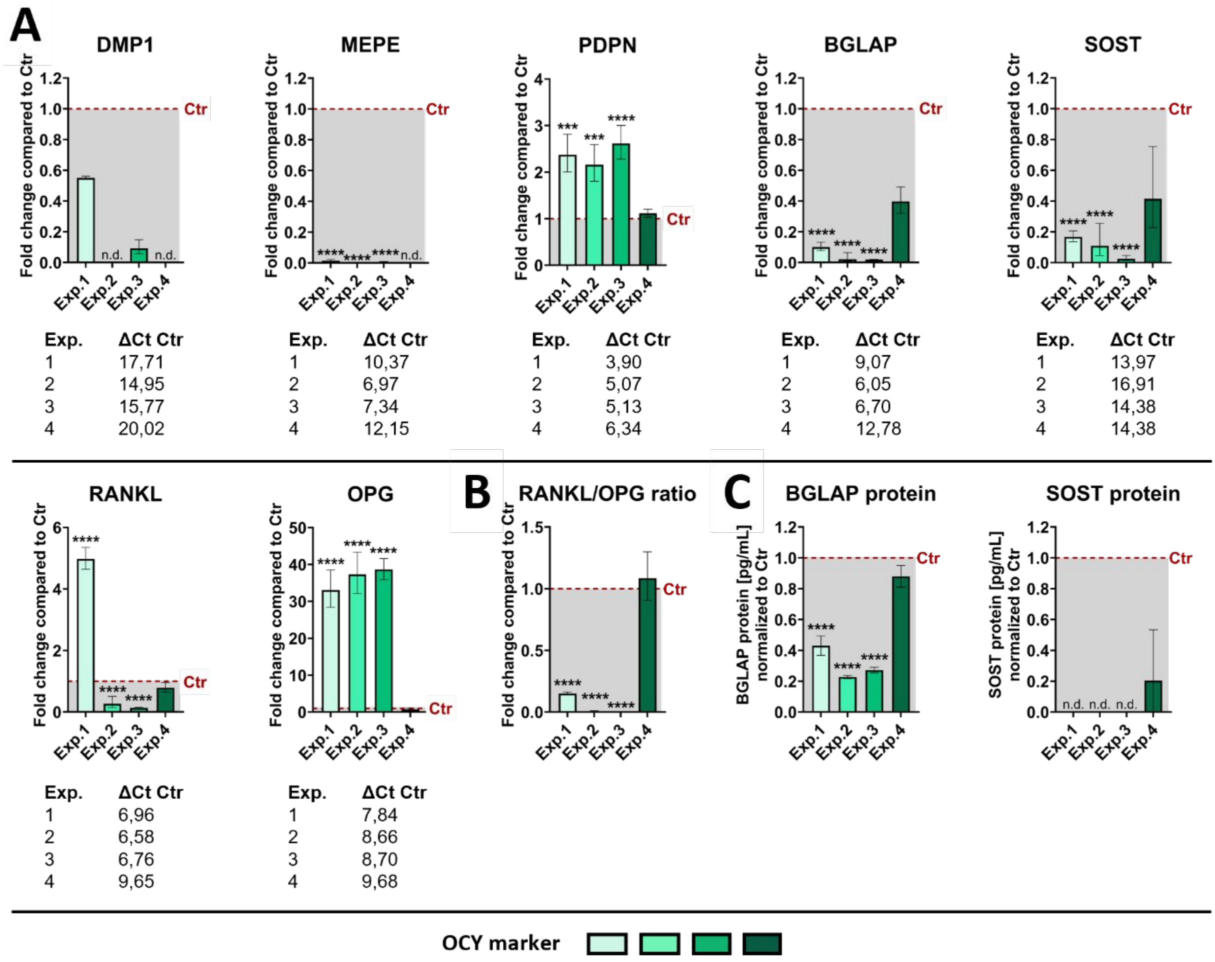

Figure S3: Impact of Mo on OCY in four individual quadruple cultures experiments with different donor combinations after 14 days treatment. (A) Gene expression of OCY markers presented as fold change normalized to Ctr (each n = 4), as well as ΔCt values of OCY in control groups. (B) RANKL/OPG ratio of OCY on gene expression level normalized to Ctr (each n = 4). (C) BGLAP and SOST protein concentrations in quadruple culture supernatants normalized to Ctr (each n = 3). ****$p < 0.0001$. n.d. not detectable.

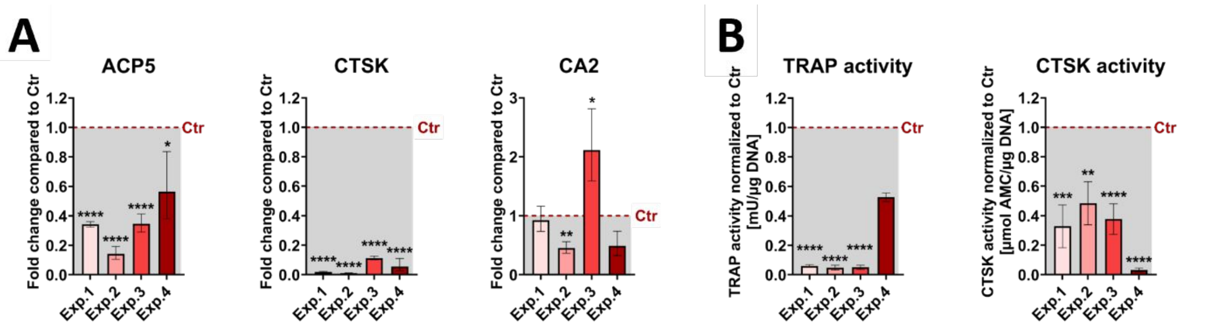

Exp.	ΔCt Ctr
1	1,48
2	1,12
3	2,14
4	6,28

Exp.	ΔCt Ctr
1	-0,26
2	0,22
3	3,22
4	5,29

Exp.	ΔCt Ctr
1	3,81
2	4,02
3	6,09
4	5,65

OC marker

Figure S4: Impact of Mo on OC in four individual quadruple cultures experiments with different donor combinations after 14 days treatment. (A) Gene expression of OC markers presented as fold change normalized to Ctr (each n = 4), as well as ΔCt values of OC in control groups. (B) OC specific enzyme activities normalized to Ctr (each n = 3). *p < 0.05; **p < 0.01; ***p < 0.001; ****p < 0.0001.

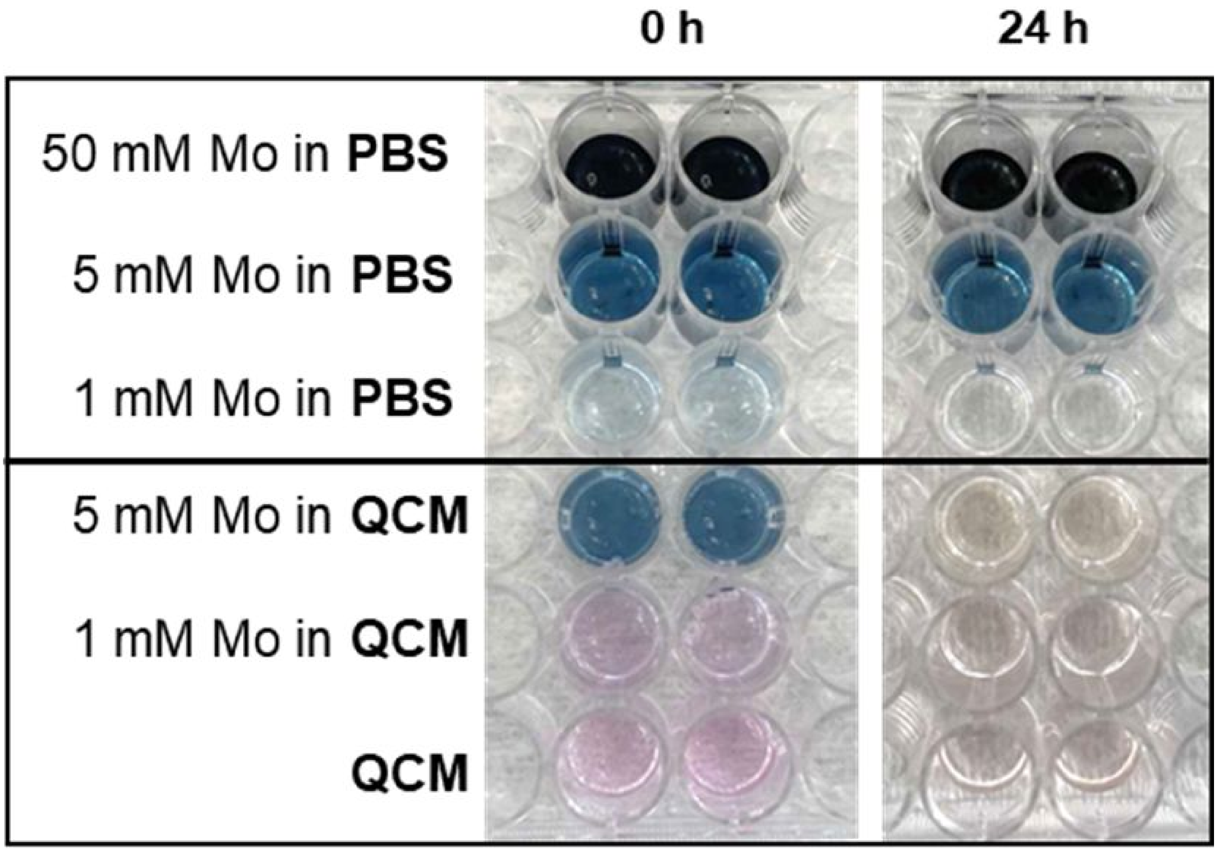

Figure S5: Mo extracts diluted in PBS or QCM directly after dilution and after 24 h incubation at 37 °C. Quadruple culture medium (QCM).
